# Supplementary material for: Functional genomics uncovers the transcription factor BNC2 as required for myofibroblastic activation in fibrosis
Source: Nat Commun. 2022 Sep 10;13:5324. doi: 10.1038/s41467-022-33063-9 (PMC9464213; doi:10.1038/s41467-022-33063-9)
Supplement: Supplementary file 1 — Supplementary information [file 41467_2022_33063_MOESM1_ESM.pdf]

Functional genomics uncovers the transcription factor BNC2 as required for myofibroblastic activation in fibrosis

SUPPLEMENTARY INFORMATION

Supplementary Figures
Supplementary References

SUPPLEMENTARY FIGURES

Fig.S1

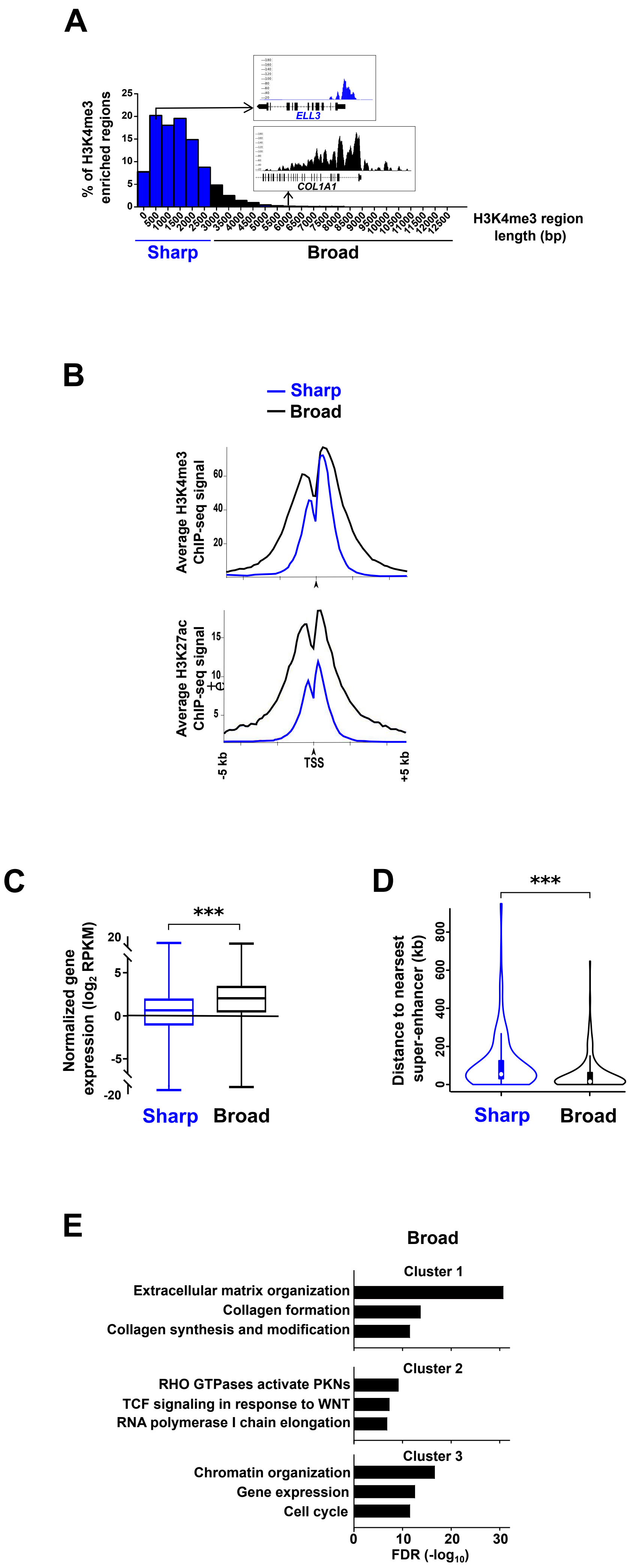
A
% of H3K4me3 enriched regions
25
20
15
10
5
0
ELL3
COL1A1
0 500 1000 1500 2000 2500 3000 3500 4000 4500 5000 5500 6000 6500 7000 7500 8000 8500 9000 9500 10000 10500 11000 11500 12000 12500
H3K4me3 region length (bp)
Sharp
Broad
B
Sharp
Broad
Average H3K4me3 ChIP-seq signal
60
40
20
Average H3K27ac ChIP-seq signal
15
10
5
-5 kb
TSS
+5 kb
C
Normalized gene expression (log2 RPKM)
***
20
5
0
-5
-20
Sharp
Broad
D
Distance to nearsest super-enhancer (kb)
***
800
600
400
200
0
Sharp
Broad
E
Broad
Cluster 1
Extracellular matrix organization
Collagen formation
Collagen synthesis and modification
Cluster 2
RHO GTPases activate PKNs
TCF signaling in response to WNT
RNA polymerase I chain elongation
Cluster 3
Chromatin organization
Gene expression
Cell cycle
0
10
20
30
FDR (-log10)

Supplementary Fig.1. Additional characterization of MF-HSC broad H3K4me3 regions and associated genes.

A. Length distribution of called H3K4me3 regions. Bars corresponding to sharp (n=13592) and broad (n=2018) regions were colored in blue and black, respectively. H3K4me3 ChIP-seq profiles at the *ELL3* and *COL1A1* genes (insets) are shown to illustrate genes associated with a sharp or a broad H3K4me3 domain, respectively. **B.** Average MF-HSC H3K4me3 (top panel) and H3K27ac (bottom panel) ChIP-seq signal intensity around the TSS of genes associated with sharp (n=13592 genes) or broad (n=2018 genes) H3K4me3 domains. **C**. Box plots showing normalized expression levels in MF-HSCs of genes associated with sharp (n=11158 genes) or broad (n=1583 genes) H3K4me3 domains. Box plots are composed of a box from the 25th to the 75th percentile with the median as a line and min to max as whiskers. Statistical significance was assessed using two-tailed Mann-Whitney U-test. *** $p<0.0001$. **D.** The TSS of genes associated with sharp (n=13592 genes) or broad (n=2018 genes) H3K4me3 domains was used to monitor the distance to the nearest H3K27ac super-enhancer from MF-HSCs. The distributions of obtained distances are shown using violin plots. Box plots are composed of a box from the 25th to the 75th percentile with the median as a white dot and min to max as whiskers. Statistical significance was assessed using two-sided Wilcoxon signed rank test with continuity correction. *** p=2.984e-09. **E.** Enrichment analysis of Reactome pathways in genes from clusters 1-3 (defined in Fig.1B) performed using the ToppGene Suite. Top 3 terms with Benjamini–Yekutieli corrected P-values <0.001 are shown. FDR: false discovery rate.

FigS2

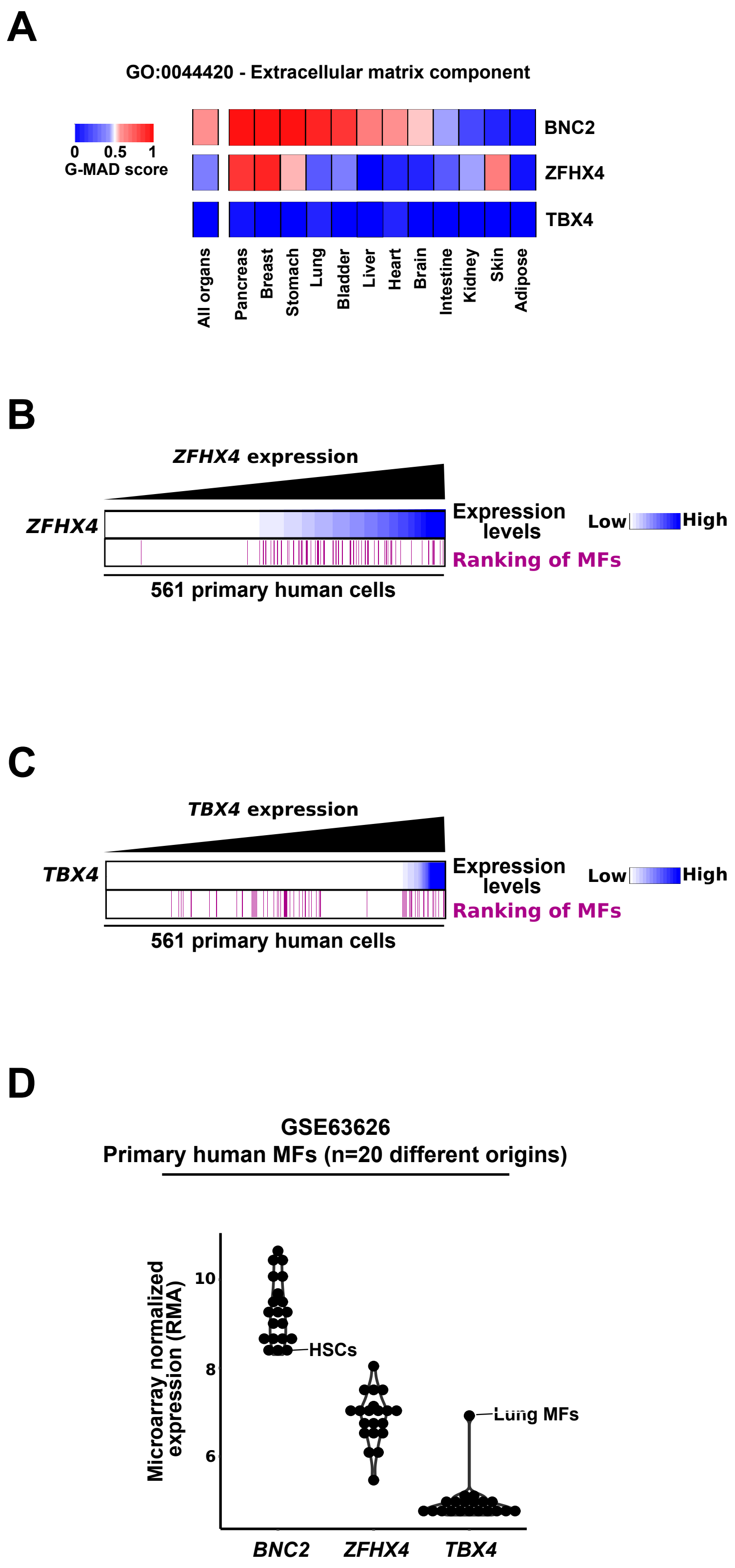
A
GO:0044420 - Extracellular matrix component
0 0.5 1
G-MAD score
BNC2
ZFHX4
TBX4
All organs
Pancreas
Breast
Stomach
Lung
Bladder
Liver
Heart
Brain
Intestine
Kidney
Skin
Adipose
B
ZFHX4 expression
ZFHX4
Expression levels
Low
High
Ranking of MFs
561 primary human cells
C
TBX4 expression
TBX4
Expression levels
Low
High
Ranking of MFs
561 primary human cells
D
GSE63626
Primary human MFs (n=20 different origins)
Microarray normalized expression (RMA)
10
8
6
HSCs
Lung MFs
BNC2
ZFHX4
TBX4

Supplementary Fig.2. *BNC2* is more widely associated with ECM gene expression across human organs than *ZFHX4* and *TBX4*.

A. Results of analyses performed using the Gene-module association Determination (G-MAD) tool used in Fig.2C. The heatmap shows G-MAD scores obtained when monitoring association of *BNC2*, *ZFHX4* or *TBX4* with GO:0044420 (ECM component) in all or 12 individual human organs. **B.** 561 primary human cells were ranked based on increasing *ZFHX4* median expression level. Ranking of 64 MF cell types is indicated at the bottom using purple horizontal bars. **C.** 561 primary human cells were ranked based on increasing *TBX4* median expression level. Ranking of 64 MF cell types is indicated at the bottom using purple horizontal bars. **D.** Normalized gene expression levels of *BNC2*, *ZFHX4* and *TBX4* in 20 primary human MFs from different origins (Colon submucosal, Colon subperitoneal, Breast dermal, Duodenum submucosal, Duodenum subperitoneal, Esophagus submucosal, Esophagus subperitoneal, Gallbladder, Hepatic stellate cells, Ileum submucosal, Ileum subperitoneal, Liver, Lung, Mammary, Mammary gland, Prostate, Stomach submucosal, Stomach subperitoneal, Uterine, Vascular adventitial). Transcriptomic data were obtained from GSE63626 [1]. Contrary to *TBX4*, which is specifically high in lung MFs, expression of *BNC2* and *ZFHX4* showed a continuum of variability around average MF expression and no specific expression in a given MF subset.

Fig.S3

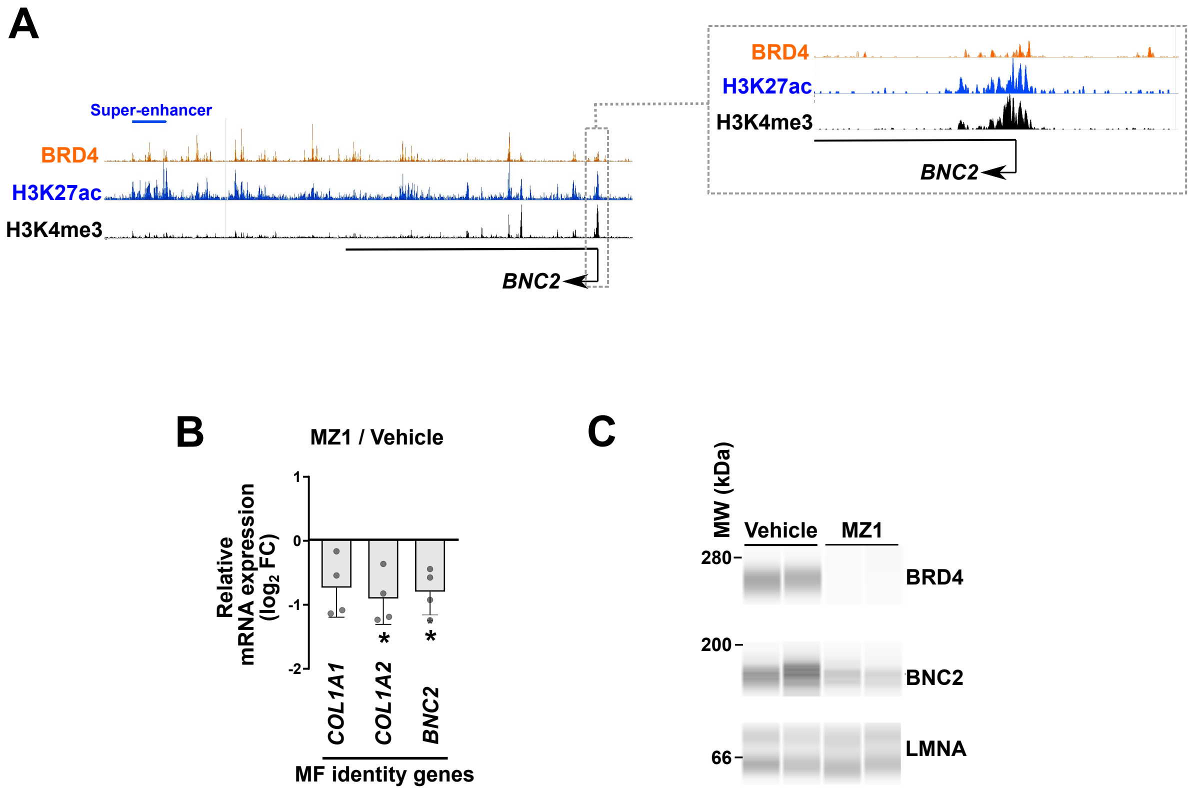
A
Super-enhancer
BRD4
H3K27ac
H3K4me3
BNC2
B
MZ1 / Vehicle
Relative mRNA expression (log2 FC)
1
0
-1
-2
COL1A1
COL1A2
BNC2
MF identity genes
C
MW (kDa)
Vehicle
MZ1
280
200
66
BRD4
BNC2
LMNA

Supplementary Fig.3. BRD4 extensively binds to and regulates *BNC2* expression in human MF-HSCs.

A. Binding of BRD4 at the *BNC2* gene. The Integrated Genome Browser (IGB) was used to visualize ChIP-seq profiles for BRD4 (orange track, LX2 cells), H3K27ac (blue track, primary MF-HSCs) and H3K4me3 (black track, primary MF-HSCs). A downstream H3K27ac super-enhancer (from Supplementary Fig.1D) is highlighted. The inset shows a zoomed view of the *BNC2* promoter region. **B.** RT-qPCR data showing the changes in expression of *BNC2*, *COL1A1* and *COL1A2* induced by the treatment of LX2 cells with 0.5 μM MZ1 for 17h (n=4 biologically independent experiments). Log_2 FC between drug and vehicle-treated cells are shown. The bar graph shows means ±SD. Two-sided one-sample t-test with Benjamini-Hochsberg correction for multiple testing was used to determine if the mean log_2 FC was statistically different from 0. * $p<0.05$, ** $p<0.01$, *** $p<0.001$. **C.** Total protein extracts from LX2 cells treated as in B were subjected to Simple Western immunoassay using antibodies directed against BRD4 and BNC2. LMNA was used as a loading control. Shown images (2 technical replicates) are representative of 2 biologically independent experiments. MW, molecular weight markers.

Fig.S4

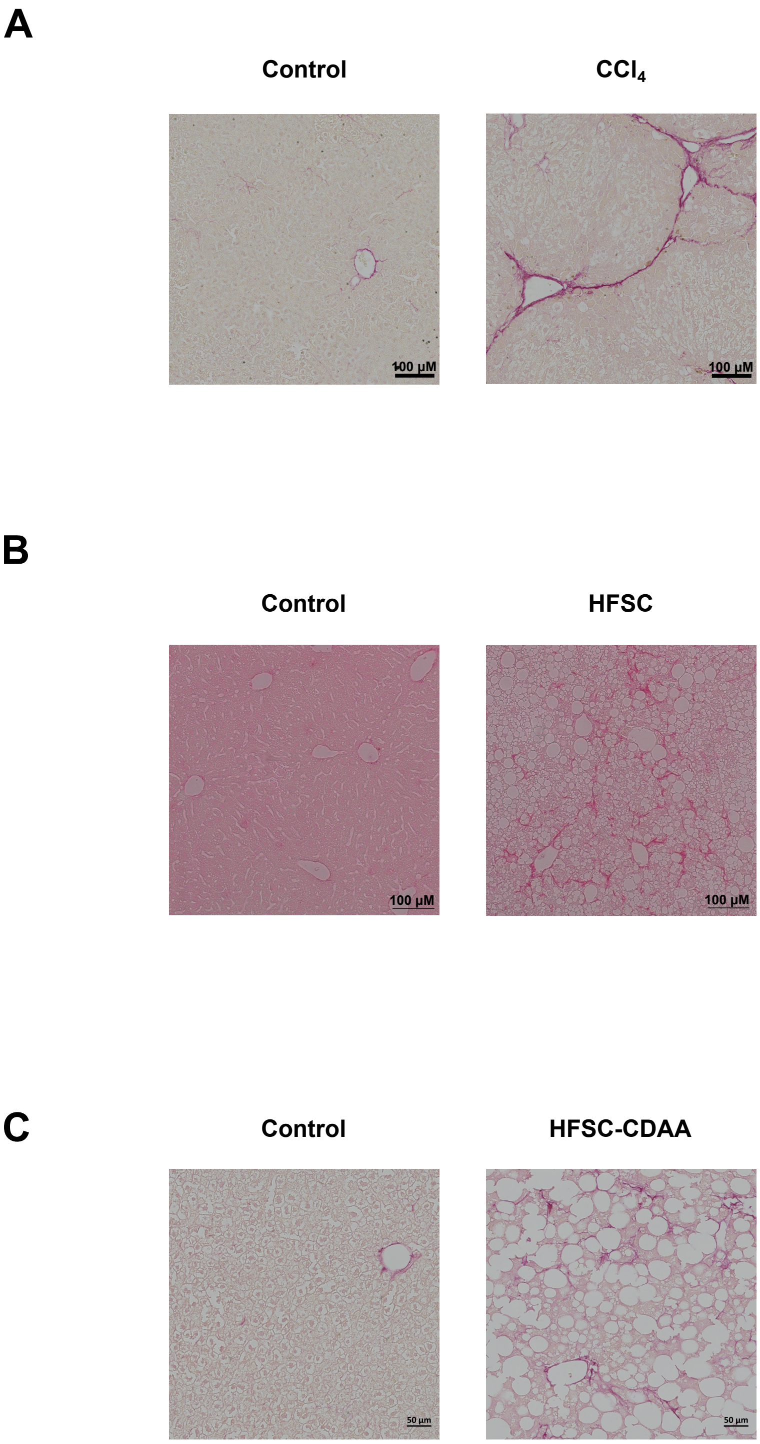

A
Control
CCl4
100 μM
100 μM
B
Control
HFSC
100 μM
100 μM
C
Control
HFSC-CDAA
50 μm
50 μm

Supplementary Fig.4. Histological validation of the mouse liver fibrosis models used in this study.

A. Representative Sirius red staining of liver sections obtained from mice injected with CCl_4 or olive oil (control) for 8 weeks (related to Fig.3A). **B.** Representative Sirius red staining of liver sections obtained from mice fed the HFSC diet or a standard rodent chow diet (control) for 24 weeks (related to Fig.3C). **C.** Representative Sirius red staining of liver sections obtained from mice fed the CDAA-HFSC diet or standard rodent chow diet (control) for 7.5 weeks (related to Fig.3D and Supplementary Fig.18). Images are representative of those obtained using liver sections from at least 10 mice per group.

Fig.S5

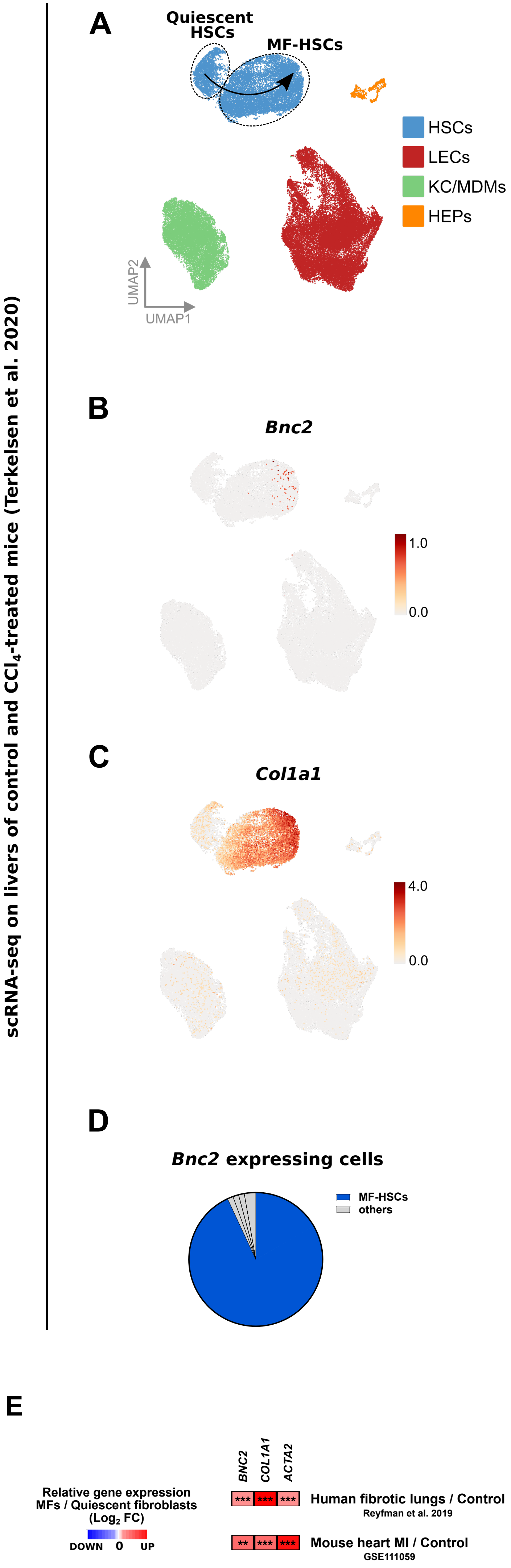

scRNA-seq on livers of control and CCl4-treated mice (Terkelsen et al. 2020)
A
Quiescent HSCs
MF-HSCs
HSCs
LECs
KC/MDMs
HEPs
UMAP2
UMAP1
B
Bnc2
1.0
0.0
C
Col1a1
4.0
0.0
D
Bnc2 expressing cells
MF-HSCs
others
E
BNC2
COL1A1
ACTA2
Relative gene expression
MFs / Quiescent fibroblasts
(Log2 FC)
DOWN
0
UP
*** *** ***
Human fibrotic lungs / Control
Reyfman et al. 2019
** *** ***
Mouse heart MI / Control
GSE111059

Supplementary Fig.5. *Bnc2* expression in mouse and human MFs from fibrotic tissues

A. Mice were treated for 2 weeks or 4 weeks with CCl_4 or with a vehicle (control) and livers were used for single-cell RNA-seq analyses [2]. UMAP plot showing 48000 cells clustered in 4 major liver cell populations. HSCs: hepatic stellate cells, LECs: liver endothelial cells, KC/MDMs: kupffer cells and monocyte-derived macrophages. Quiescent and activated HSCs (MF-HSCs) were further highlighted based on in-depth analysis of the data previously performed [2]. **B-C.** Single-cell expression of *Bnc2* and *Col1a*1 on the UMAP plot described in A. Log_2 expression is shown as overlay (n=3). As typically observed for TFs, *Bnc2* expression was more scarcely detected than that of a non-TF encoding gene such as *Col1a1*. **D.** Pie chart showing the distribution of *Bnc2* expressing cells in the different liver cell populations: CCl_4 MF-HSCs (n=67 cells); control LSEC (n=1 cell), CCl_4 LSECs (n=2 cells), CCl_4 KC/MDMs (n=2 cells). **E.** Heatmap showing Log_2 FC of *BNC2*, *COL1A1* and *ACTA2* expression in MFs isolated from fibrotic organs relative to the expression in quiescent fibroblasts (i.e. isolated from non-fibrotic organs). Human fibrotic lung MF transcriptomic data were obtained from [3]. MFs from mouse hearts subjected to myocardial infarction (MI) were obtained from GSE111059 [4]. False discovery rates (FDR) issued from the transcriptomic analyses are shown.

Fig.S6

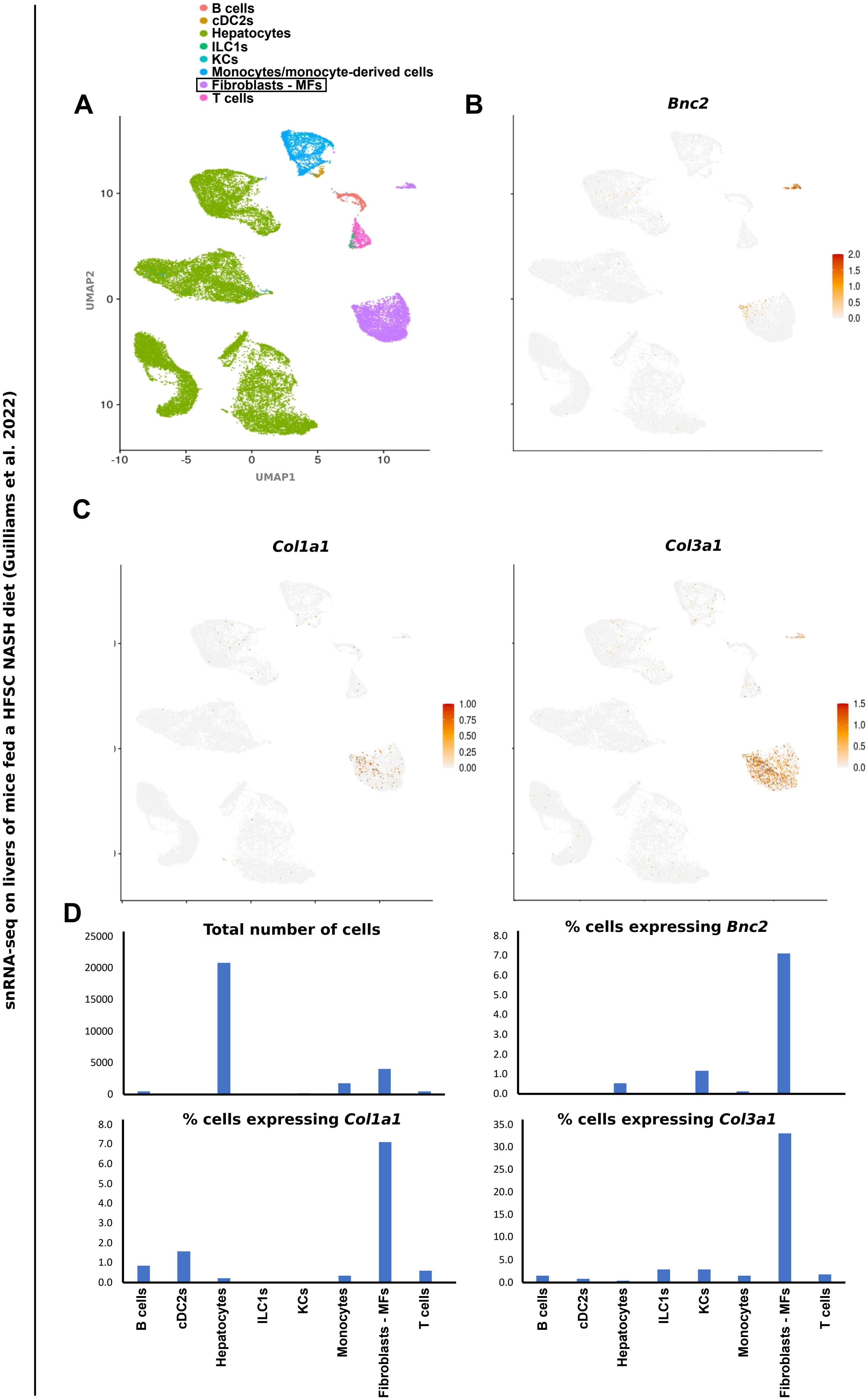
snRNA-seq on livers of mice fed a HFSC NASH diet (Guilliams et al. 2022)
A
B cells
cDC2s
Hepatocytes
ILC1s
KCs
Monocytes/monocyte-derived cells
Fibroblasts - MFs
T cells
UMAP1
UMAP2
B
Bnc2
C
Col1a1
Col3a1
D
Total number of cells
% cells expressing Bnc2
% cells expressing Col1a1
% cells expressing Col3a1
B cells
cDC2s
Hepatocytes
ILC1s
KCs
Monocytes
Fibroblasts - MFs
T cells

Supplementary Fig.6. *Bnc2* expression in liver cells defined using single-nuclei RNA-seq analysis of mice fed a HFSC NASH diet.

A. Mice were fed a HFSC diet for 24 or 36 weeks and livers were used for single-nuclei RNA-seq analyses [5]. UMAP plot showing the 8 main clusters of liver cell populations annotated according to [5]. cDC, conventional dendritic cells; ILC, Innate lymphoid cells; KC : kupffer cells. **B-C.** Single-nuclei expression of *Bnc2*, *Col1a*1 and *Col3a1* on the UMAP plot described in A. Normalized $\log_2$ expression is shown as overlay. **D.** Fraction of each liver cell-type expressing detectable *Bnc2*, *Col1a1* or *Col3a1* levels in the single-nuclei data.

Fig.S7

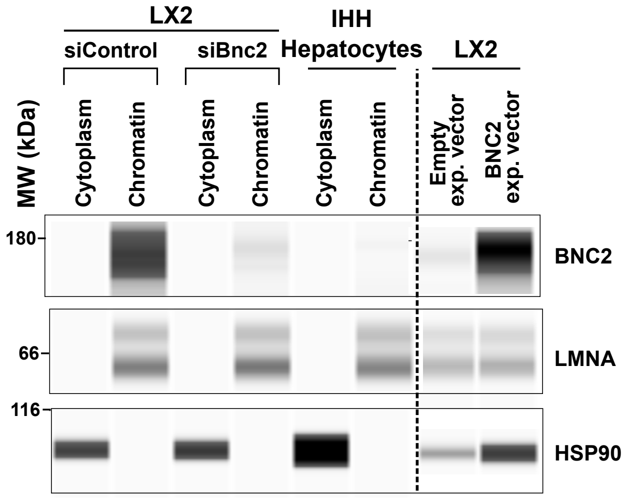
LX2
siControl
siBnc2
IHH
Hepatocytes
LX2
MW (kDa)
Cytoplasm
Chromatin
Cytoplasm
Chromatin
Cytoplasm
Chromatin
Empty
exp. vector
BNC2
exp. vector
180
66
116
BNC2
LMNA
HSP90

Supplementary Fig.7. BNC2 antibody specificity in immunoblotting experiments.

LX2 cells were transfected with a siRNA targeting BNC2 (siBNC2) or a non-targeting control siRNA (siCTRL) on the one hand, and with an expression vector (exp. vector) encoding human BNC2 or an empty vector (control) on the other hand. IHH human hepatocytes served as control cells devoid of *BNC2* expression. Total cellular extracts or sub-cellular fractions (cytoplasm and chromatin fractions) were used in Simple Western immunoassays using an antibody directed against BNC2 (Sigma, HPA059419). LMNA and HSP90 were used as controls of efficient sub-cellular fractionation and equal loading. BNC2 silencing is representative of at least 4 biologically independent replicates. MW, molecular weight markers.

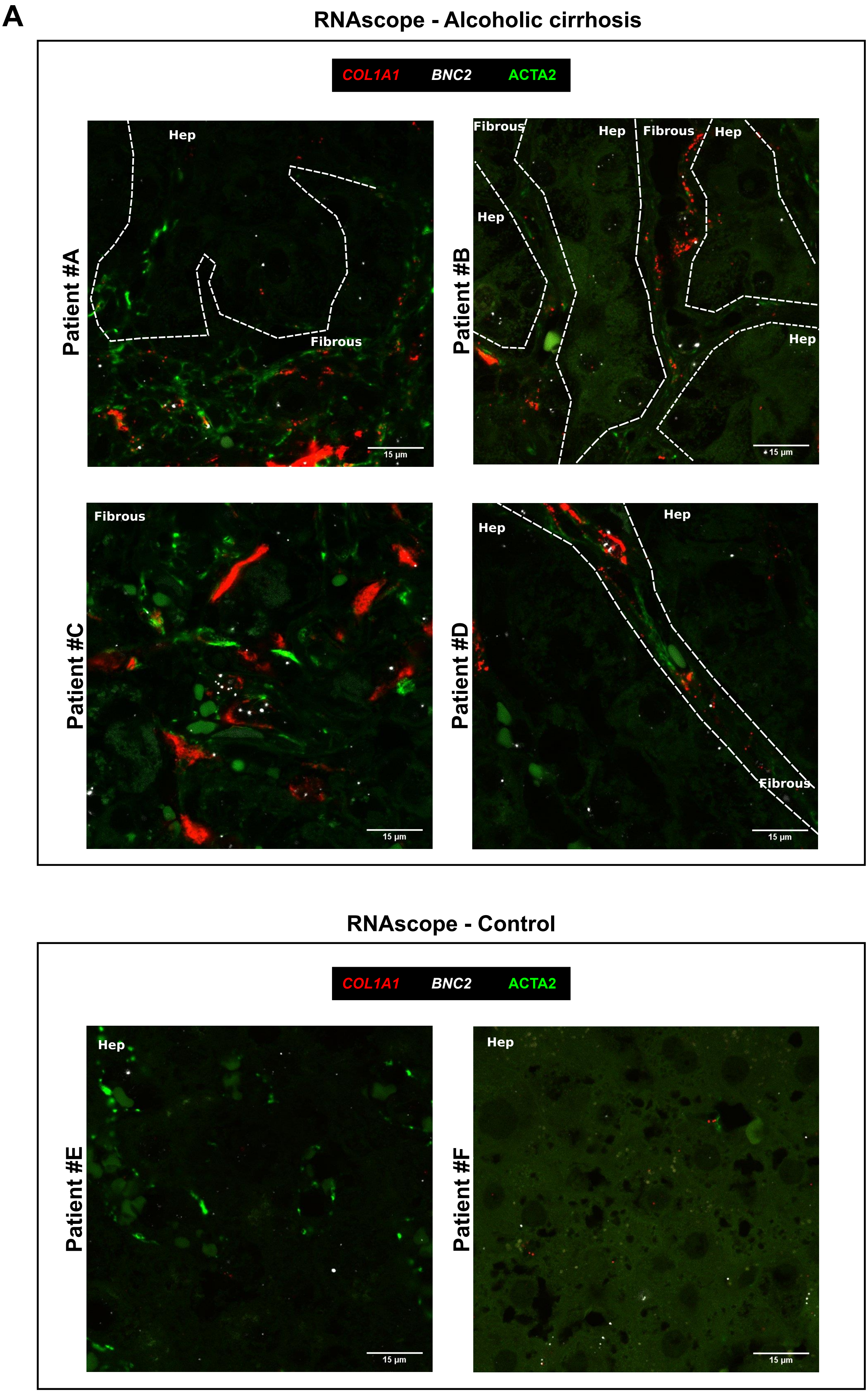
A
RNAscope - Alcoholic cirrhosis
COL1A1
BNC2
ACTA2
Patient #A
Hep
Fibrous
15 µm
Patient #B
Fibrous
Hep
Fibrous
Hep
Hep
Hep
15 µm
Patient #C
Fibrous
15 µm
Patient #D
Hep
Hep
Fibrous
15 µm
RNAscope - Control
COL1A1
BNC2
ACTA2
Patient #E
Hep
15 µm
Patient #F
Hep
15 µm

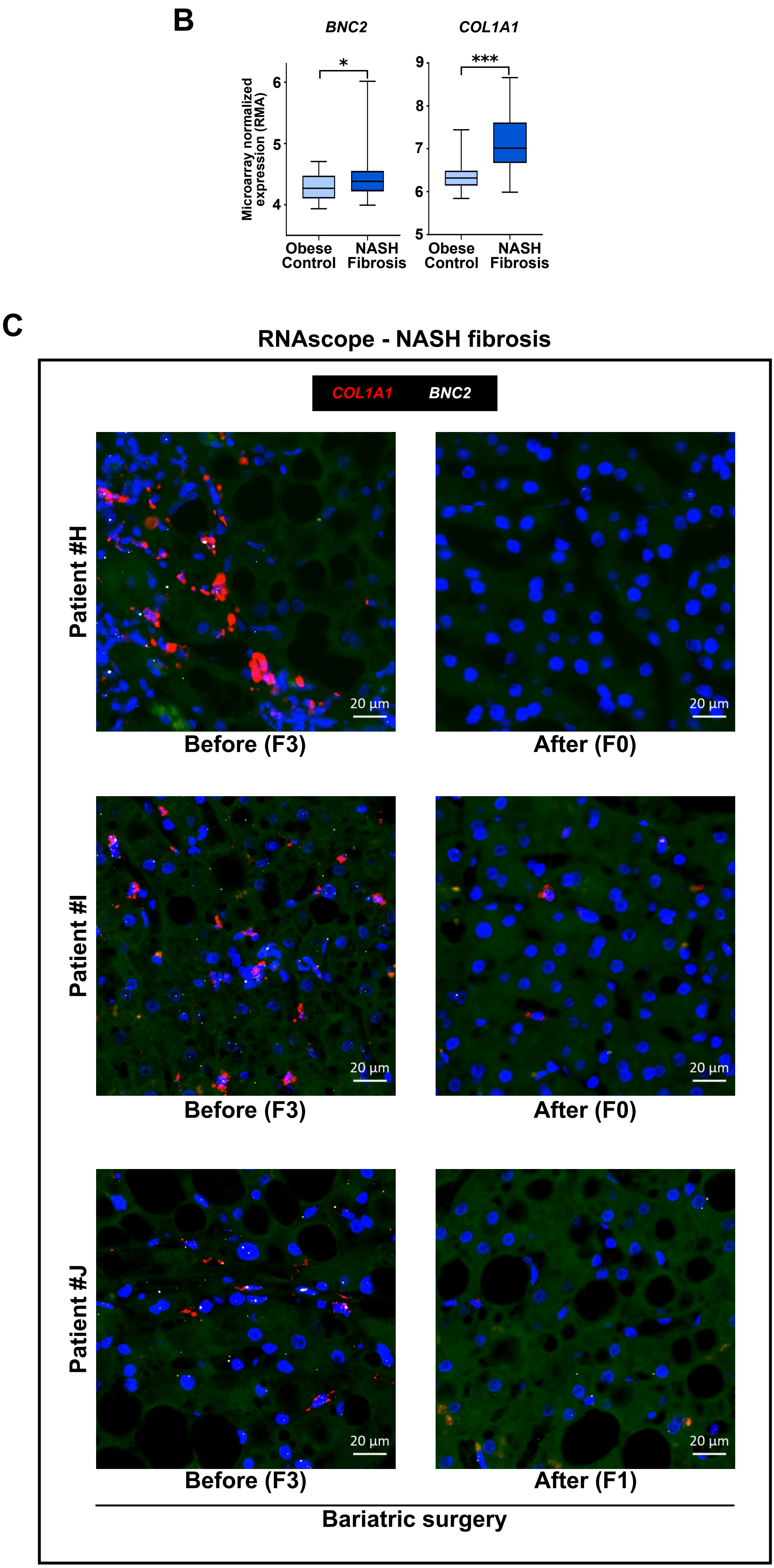
B
BNC2
COL1A1
Microarray normalized expression (RMA)
*
***
6
5
4
9
8
7
6
5
Obese Control
NASH Fibrosis
Obese Control
NASH Fibrosis
C
RNAscope - NASH fibrosis
COL1A1
BNC2
Patient #H
Patient #I
Patient #J
20 µm
Before (F3)
After (F0)
Before (F3)
After (F0)
Before (F3)
After (F1)
Bariatric surgery

Supplementary Fig.8. Additional *In situ* RNA hybridization images of human liver sections related to Fig.4C and Fig.4D.

A. RNAscope analyses were performed on paraffin-embedded liver sections obtained from patients presenting alcohol-related cirrhosis (n=4; a different field from the one shown in Fig.4C is provided for patient #A) or control samples from the non-tumoral areas of liver resections (n=2). Oligonucleotide probes were designed against *BNC2* (white staining) or *COL1A1* (red staining) transcripts. Immunofluorescence staining was further performed for the detection of ACTA2 protein (green staining). Dotted lines delimitate the hepatocyte and the fibrous areas.

B. Normalized *BNC2* and *COL1A1* expression levels obtained from the transcriptomic analyses of human liver biopsies from patients with advanced NASH-associated fibrosis (Kleiner Score F3-4) and morbidly obese controls without liver fibrosis (Kleiner Score F0) matched for potential confounders (n=53 biologically independent samples per group) based on our previous analyses of this cohort [6]. Box plots are composed of a box from the 25th to the 75th percentile with the median as a white dot and min to max as whiskers. Statistical significance was assessed using two-tailed Mann-Whitney U-test. * p=0.0391, *** p<0.0001.

C. RNAscope analyses were performed as described in Fig.4D on paraffin-embedded liver biopsies of additional morbidly obese NASH patients showing bariatric surgery induced loss of fibrosis (Kleiner Score F3 before surgery to F0/1 one or 5 years after surgery). Oligonucleotide probes were designed against BNC2 (white staining) or COL1A1 (red staining) transcripts. Quantifications of signals given by the *BNC2* and *COL1A1* oligonucleotide probes (n=4 biologically independent sets of samples) are shown in Fig.4D.

Fig.S9

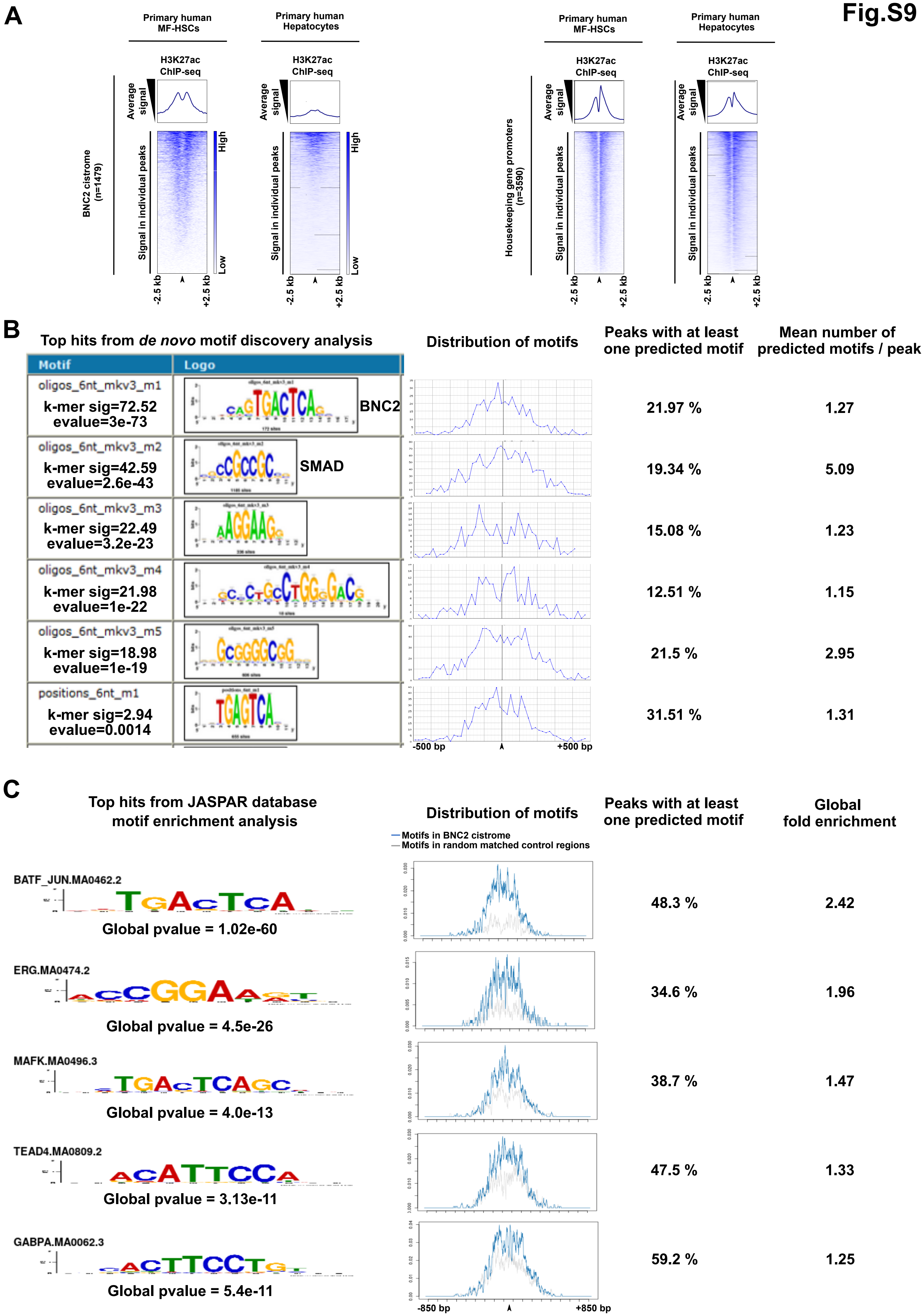
A
Primary human MF-HSCs
Primary human Hepatocytes
H3K27ac ChIP-seq
Average signal
Signal in individual peaks
High
Low
-2.5 kb
+2.5 kb
BNC2 cistrome (n=1479)
Housekeeping gene promoters (n=3590)
B
Top hits from de novo motif discovery analysis
Distribution of motifs
Peaks with at least one predicted motif
Mean number of predicted motifs / peak
Motif
Logo
oligos_6nt_mkv3_m1
k-mer sig=72.52
evalue=3e-73
BNC2
21.97 %
1.27
oligos_6nt_mkv3_m2
k-mer sig=42.59
evalue=2.6e-43
SMAD
19.34 %
5.09
oligos_6nt_mkv3_m3
k-mer sig=22.49
evalue=3.2e-23
15.08 %
1.23
oligos_6nt_mkv3_m4
k-mer sig=21.98
evalue=1e-22
12.51 %
1.15
oligos_6nt_mkv3_m5
k-mer sig=18.98
evalue=1e-19
21.5 %
2.95
positions_6nt_m1
k-mer sig=2.94
evalue=0.0014
31.51 %
1.31
-500 bp
+500 bp
C
Top hits from JASPAR database motif enrichment analysis
Distribution of motifs
Motifs in BNC2 cistrome
Motifs in random matched control regions
Peaks with at least one predicted motif
Global fold enrichment
BATF_JUN.MA0462.2
Global pvalue = 1.02e-60
48.3 %
2.42
ERG.MA0474.2
Global pvalue = 4.5e-26
34.6 %
1.96
MAFK.MA0496.3
Global pvalue = 4.0e-13
38.7 %
1.47
TEAD4.MA0809.2
Global pvalue = 3.13e-11
47.5 %
1.33
GABPA.MA0062.3
Global pvalue = 5.4e-11
59.2 %
1.25
-850 bp
+850 bp

Fig.S9

D

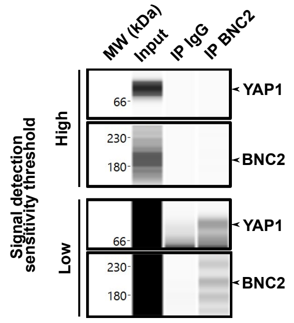
MW (kDa)
Input
IP IgG
IP BNC2
Signal detection sensitivity threshold
High
Low
66
230
180
YAP1
BNC2

E

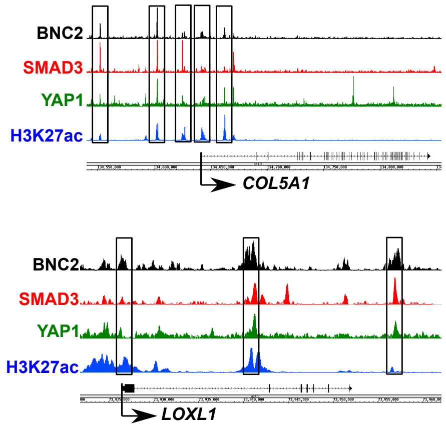
BNC2
SMAD3
YAP1
H3K27ac
COL5A1
LOXL1

F

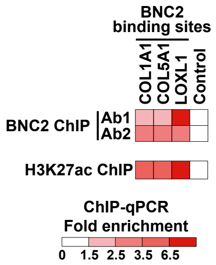
BNC2 binding sites
COL1A1
COL5A1
LOXL1
Control
BNC2 ChIP
Ab1
Ab2
H3K27ac ChIP
ChIP-qPCR Fold enrichment
0
1.5
2.5
3.5
6.5

Supplementary Fig.9. BNC2 cistrome additional characterization.

A. H3K27ac ChIP-seq signals from human primary MF-HSCs or hepatocytes were visualized at the BNC2 cistrome (1479 binding sites; top heatmaps). The TSS of housekeeping genes (n=3590 genes; bottom heatmaps) were used as positive controls showing H3K27ac labelling in both MF-HSCs and hepatocytes. Heatmaps show the signals in 5 kb regions centered on the BNC2 peaks or gene TSS. Average signals are plotted on top of the heatmaps. **B.** *De novo* motif enrichment analyses performed using the BNC2 cistrome and RSAT. Top enriched motifs (e-value<0.005) are shown. While the first motif matches the proposed BNC2 recognition motif [7], the second motif is GC-rich and could promote, among others, the binding of SMAD TFs [8]. **C.** Top 5 TF DNA recognition motifs from the JASPAR CORE 2020 database enriched within the BNC2 cistrome identified by TFmotifView. **D.** Nuclear extracts from formaldehyde cross-linked LX2 cells were subjected to immunoprecipitation with an antibody against BNC2 (HPA018525, Sigma-Aldrich). Immunoprecipitated material was analyzed by Western Blot or Simple western using antibodies directed against BNC2 or YAP1. Shown images are representative of those obtained for 2 biologically independent replicates. MW, molecular weight markers. **E.** The Integrated Genome Browser (IGB) was used to visualize ChIP-seq profiles for BNC2 (black track, LX2 cells), SMAD3 (red track, LX2 cells), YAP1 (green track, IMR90 cells) and H3K27ac (blue track, MF-HSCs) at the *COL5A1* and *LOXL1* genes **F.** ChIP-qPCR validation of BNC2 binding at the *COL1A1*, *COL5A1* and *LOXL1* genes in LX2 cells. Two antibodies against BNC2 were used in these assays. The first antibody (Ab1; Sigma, HPA059419) was different from the one used for ChIP-seq (Ab2; Sigma, HPA018525). H3K27ac ChIP-seq was performed in parallel on the same chromatin preparations. The heatmap depicts the level of fold enrichment relative to control regions, i.e. average signal at genomic regions not bound by BNC2 used as a reference (Control) (n=2 biologically independent experiments).

Fig.S10

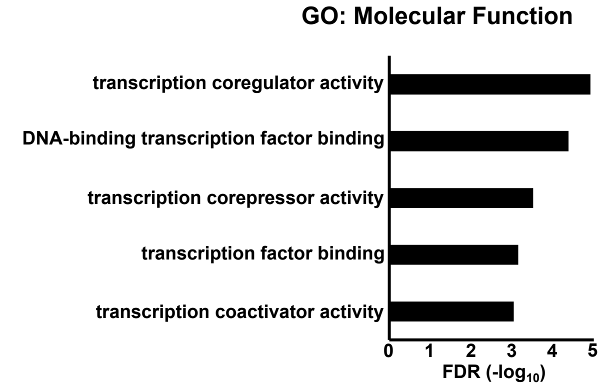

GO: Molecular Function
transcription coregulator activity
DNA-binding transcription factor binding
transcription corepressor activity
transcription factor binding
transcription coactivator activity
0
1
2
3
4
5
FDR (-log10)

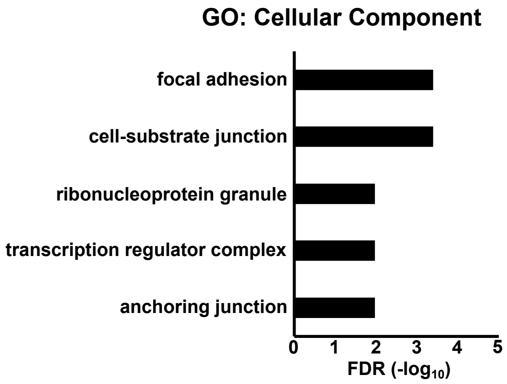

GO: Cellular Component
focal adhesion
cell-substrate junction
ribonucleoprotein granule
transcription regulator complex
anchoring junction
0
1
2
3
4
5
FDR (-log10)

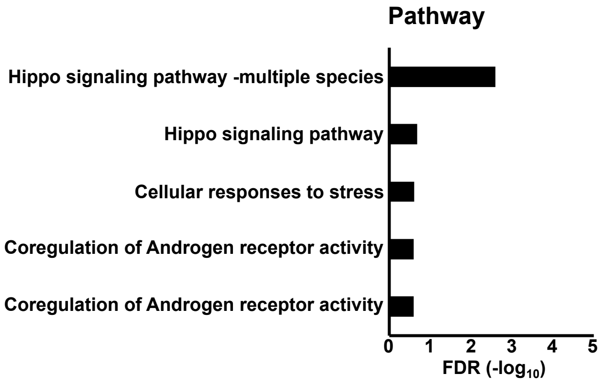

Pathway
Hippo signaling pathway -multiple species
Hippo signaling pathway
Cellular responses to stress
Coregulation of Androgen receptor activity
Coregulation of Androgen receptor activity
0
1
2
3
4
5
FDR (-log10)

Supplementary Fig.10. Characterization of BNC2 interactors identified by RIME

BNC2 interactors from Fig.5D were used for enrichment analyses with the ToppGene Suite. Top 5 terms with Benjamini–Yekutieli corrected P-values are shown. FDR: false discovery rate.

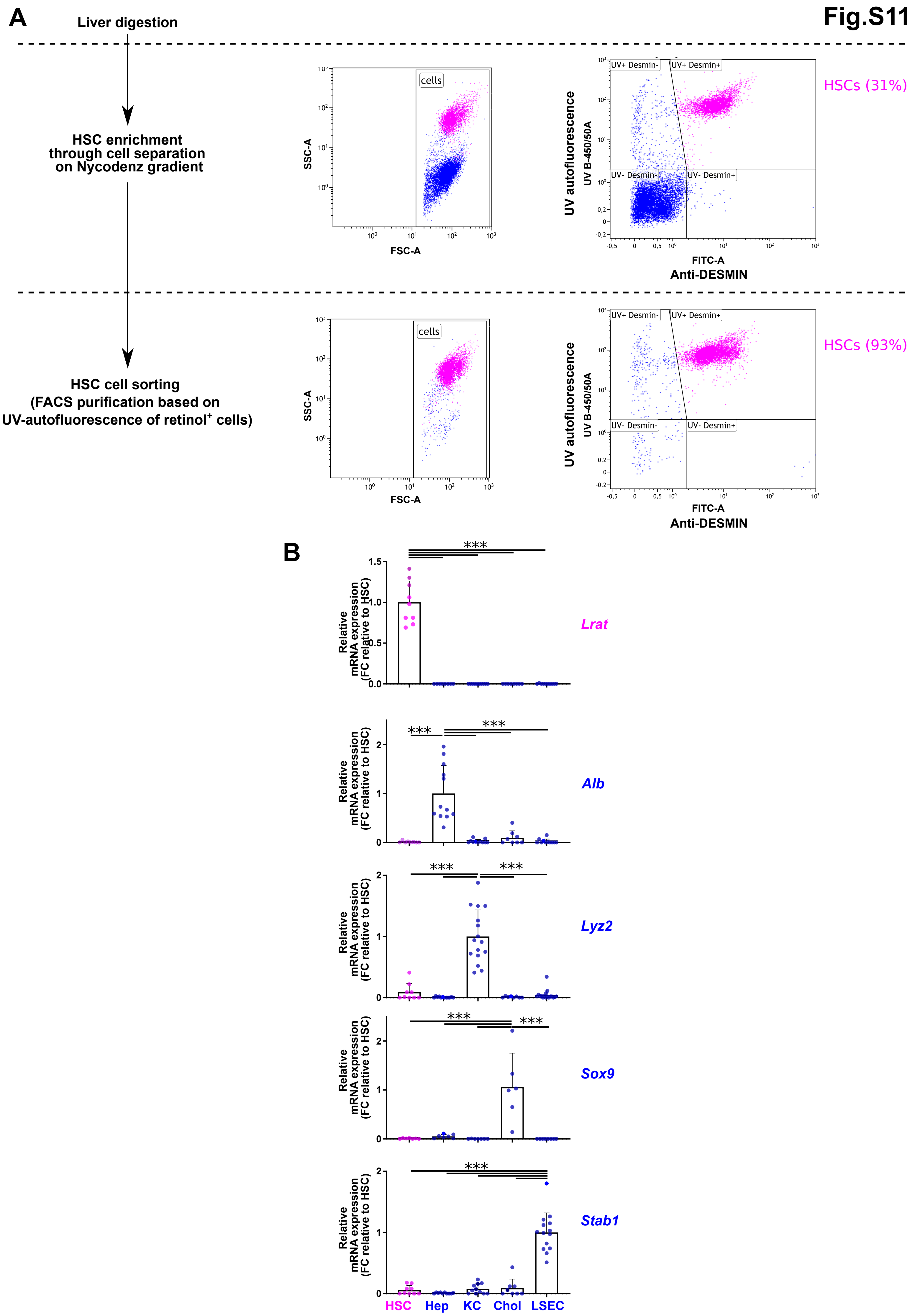
Fig.S11
A
Liver digestion
HSC enrichment through cell separation on Nycodenz gradient
HSC cell sorting (FACS purification based on UV-autofluorescence of retinol+ cells)
cells
SSC-A
FSC-A
UV autofluorescence
UV B-450/50A
UV+ Desmin-
UV+ Desmin+
UV- Desmin-
UV- Desmin+
FITC-A
Anti-DESMIN
HSCs (31%)
HSCs (93%)
B
Relative mRNA expression (FC relative to HSC)
Lrat
Alb
Lyz2
Sox9
Stab1
***
HSC
Hep
KC
Chol
LSEC

Supplementary Fig.11. Purity of sorted primary mouse HSCs and other murine liver cell types.

A. The left part of this panel provides the main successive steps of our mouse primary HSC isolation protocol. HSC purity was assessed after enrichment through Nycodenz gradient or after final cell sorting by FACS based on UV autofluorescence. Purity was defined as the % of UV and Desmin positive cells (UV+, Desmin+), which is indicated at the top right of the FACS plots. **B.** Simultaneous isolation of liver cell types was performed through liver enzymatic digestion and FACS sorting. The expression of liver cell type-specific markers *Lrat*, *Alb*, *Lyz2*, *Sox9* and *Stab1* was assessed by RT-qPCR to confirm their enrichment in HSC, hepatic stellate cells; Hep., hepatocytes; KC, Kupffer cells; Chol., cholangiocytes and LSEC, liver sinusoidal endothelial cells, respectively. Data are from biologically independent cell isolations: *Lrat* [HSC (n=9), Hep (n=8), KC (n=10), Chol (n=8), LSEC (n=10)]; *Alb* [HSC (n=8), Hep (n=12), KC (n=11), Chol (n=8), LSEC (n=11)]; *Lyz2* [HSC (n=9), Hep (n=12), KC (n=16), Chol (n=8), LSEC (n=17)]; *Sox9* [HSC (n=7), Hep (n=7), KC (n=7), Chol (n=6), LSEC (n=8)]; *Stab1* [HSC (n=9), Hep (n=10), KC (n=11), Chol (n=8), LSEC (n=14)]. The bar graphs show means ±SD. Statistical significance was assessed using one-way Anova with Tukey multiple comparison post-hoc test. *** $p<0.001$.

Fig.S12

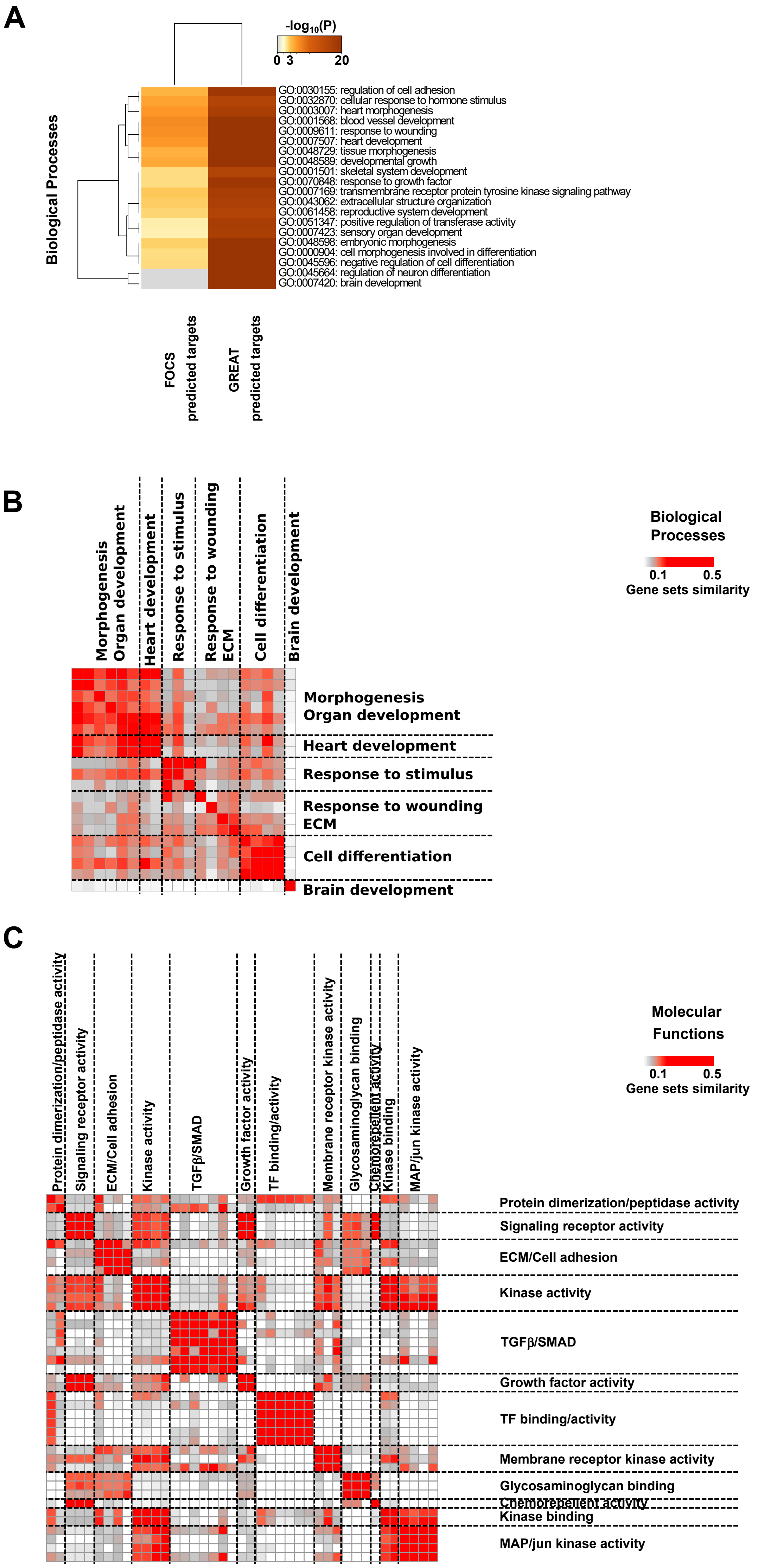
A
-log10(P)
0 3 20
Biological Processes
GO:0030155: regulation of cell adhesion
GO:0032870: cellular response to hormone stimulus
GO:0003007: heart morphogenesis
GO:0001568: blood vessel development
GO:0009611: response to wounding
GO:0007507: heart development
GO:0048729: tissue morphogenesis
GO:0048589: developmental growth
GO:0001501: skeletal system development
GO:0070848: response to growth factor
GO:0007169: transmembrane receptor protein tyrosine kinase signaling pathway
GO:0043062: extracellular structure organization
GO:0061458: reproductive system development
GO:0051347: positive regulation of transferase activity
GO:0007423: sensory organ development
GO:0048598: embryonic morphogenesis
GO:0000904: cell morphogenesis involved in differentiation
GO:0045596: negative regulation of cell differentiation
GO:0045664: regulation of neuron differentiation
GO:0007420: brain development
FOCS predicted targets
GREAT predicted targets
B
Morphogenesis
Organ development
Heart development
Response to stimulus
Response to wounding
ECM
Cell differentiation
Brain development
Biological Processes
0.1 0.5
Gene sets similarity
C
Protein dimerization/peptidase activity
Signaling receptor activity
ECM/Cell adhesion
Kinase activity
TGFβ/SMAD
Growth factor activity
TF binding/activity
Membrane receptor kinase activity
Glycosaminoglycan binding
Chemorepellent activity
Kinase binding
MAP/jun kinase activity
Molecular Functions
0.1 0.5
Gene sets similarity

Supplementary Fig.12. Detailed results of enrichment analyses of BNC2 target genes.

A. Enrichment analysis for genes involved in specific biological processes was performed using BNC2 predicted target genes based on its cistrome and Metascape. BNC2 potential target genes were defined by assigning BNC2 bound regions to proximal genes using GREAT [9] (GREAT predicted targets) or taking into account FOCS predicted promoter-enhancer interactions [10] (FOCS predicted targets). The top 20 enriched terms, clustered based on P values, are displayed. Gray color indicates a lack of significance. **B.** Biological processes identified in panel A were grouped based on gene sets similarity. 6 main clusters delineated by doted lines were identified and provided with summarizing names (see Supplementary Data file 9 for details), which are reported in Fig.7A. **C.** Enrichment analysis for genes involved in specific molecular functions was performed using genes defined as both bound and regulated by BNC2 using ToppGene. Molecular functions identified were grouped based on gene sets similarity. 12 main clusters delineated by doted lines were identified and provided with a summarizing name (see Supplementary Data file 9 for details).

Fig.S13

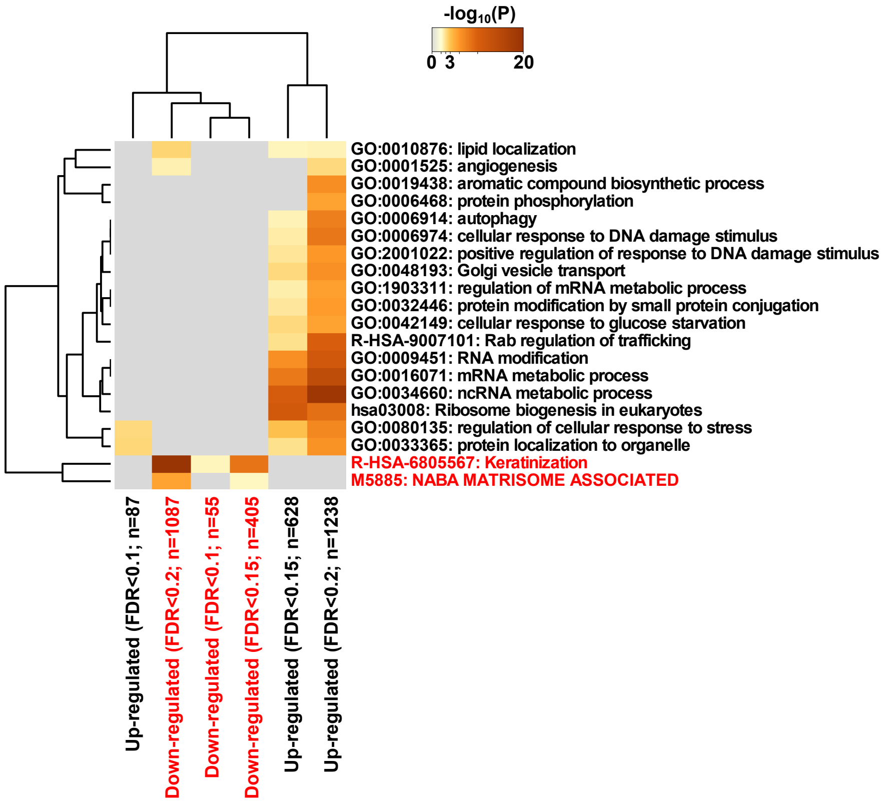

-log10(P)
0 3 20
GO:0010876: lipid localization
GO:0001525: angiogenesis
GO:0019438: aromatic compound biosynthetic process
GO:0006468: protein phosphorylation
GO:0006914: autophagy
GO:0006974: cellular response to DNA damage stimulus
GO:2001022: positive regulation of response to DNA damage stimulus
GO:0048193: Golgi vesicle transport
GO:1903311: regulation of mRNA metabolic process
GO:0032446: protein modification by small protein conjugation
GO:0042149: cellular response to glucose starvation
R-HSA-9007101: Rab regulation of trafficking
GO:0009451: RNA modification
GO:0016071: mRNA metabolic process
GO:0034660: ncRNA metabolic process
hsa03008: Ribosome biogenesis in eukaryotes
GO:0080135: regulation of cellular response to stress
GO:0033365: protein localization to organelle
R-HSA-6805567: Keratinization
M5885: NABA MATRISOME ASSOCIATED
Up-regulated (FDR<0.1; n=87
Down-regulated (FDR<0.2; n=1087
Down-regulated (FDR<0.1; n=55
Down-regulated (FDR<0.15; n=405
Up-regulated (FDR<0.15; n=628
Up-regulated (FDR<0.2; n=1238

Supplementary Fig.13. Additional characterization of BNC2 regulated genes in LX2 cells.

Genes modulated in LX2 cells by transfection of siBNC2 were called at the indicated FDR and separated between up- and down-regulated genes. The obtained lists were used for gene enrichment analyses in Metascape. Heatmap showing the top enrichment clusters is shown. Gray color indicates a lack of significance.

Fig.S14

A

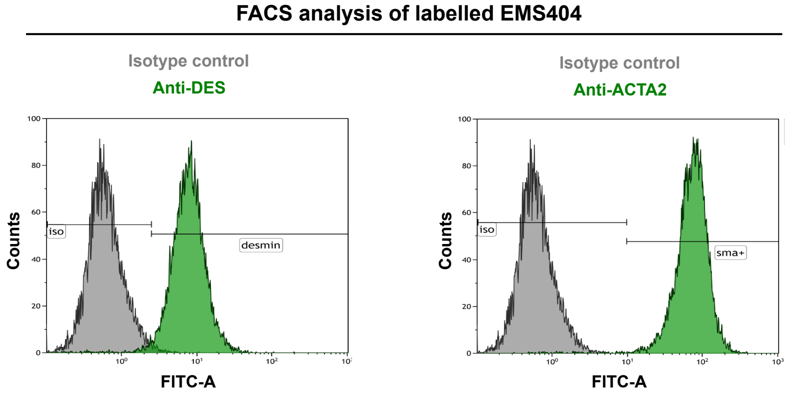

FACS analysis of labelled EMS404
Isotype control
Anti-DES
iso
desmin
Counts
FITC-A
Isotype control
Anti-ACTA2
iso
sma+
Counts
FITC-A

B

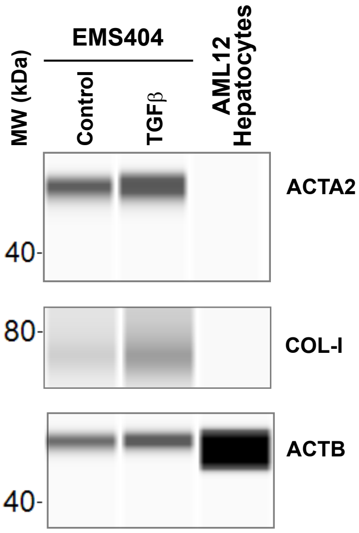

EMS404
MW (kDa)
Control
TGFβ
AML12 Hepatocytes
ACTA2
40-
180-
COL-I
ACTB
40-

Supplementary Fig.14. Characterization of the EMS404 mouse MF-HSC cell line.

A. Cells were stained with an antibody directed against DES (Desmin), ACTA2 or a non-targeting IgG (isotype control) and analyzed by FACS. Signal intensities issued from cell staining with the anti-DES and anti-ACTA2 antibodies when compared to non-immune control IgG are shown. **B.** EMS404 cells were treated for 24h with 1 ng/mL TGFβ or a vehicle (control). Total protein extracts were subjected to Simple Western immunoassay using antibodies directed against ACTA2 or type I collagen (COL-I). ACTB was used as a protein loading control. AML12 hepatocytes were used as a non-MF control cell line. MW, molecular weight markers.

Fig.S15

A

Collagens

LX2

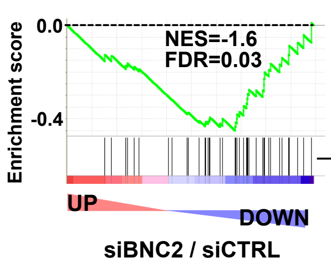

GSEA core enrichment

COL24A1
COL14A1
COL13A1
COL7A1
COL6A1
COL11A2
COL15A1
COL28A1
COL9A3
COL26A1
COL21A1
COL22A1
COL5A1
COL16A1
COL5A3
COL6A3
COL6A5
COL19A1
COL4A3
COL11A1
COL1A2
COL9A1
COL2A1
COL1A1
COL3A1

EMS404

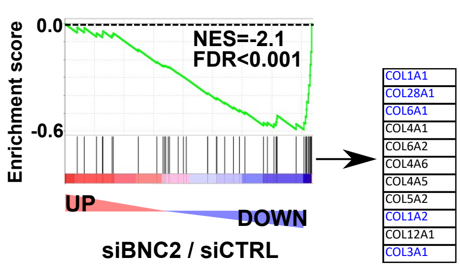

COL1A1
COL28A1
COL6A1
COL4A1
COL6A2
COL4A6
COL4A5
COL5A2
COL1A2
COL12A1
COL3A1

B

ECM regulators

LX2

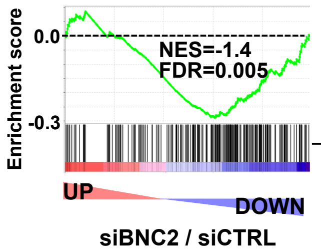

ADAMTSL5	ADAMTS3
ITIH1	PAMR1
ADAM19	CPAMD8
HYAL4	ADAM22
ITIH2	SPAM1
ITIH4	CST9L
TGM2	F2
ADAM11	ITIH6
ASTL	SERPINA12
LPA	CELA1
MMP21	MASP1
SERPINB12	ADAMTS18
F13A1	TIMP1
PCSK6	CTSB
A2M	CST6
CTSZ	HTRA4
ADAMTS17	C17orf58
MMP15	PLOD1
MEP1A	MMP17
ADAM2	CTSA
MMP16	AGT
CPN2	CTSV
ADAM8	SERPINA1
ADAM23	TGM3
SERPINI1	SERPINB8
TGM6	SERPINI2
SERPINB4	SERPINE2
PCSK5	HRG
MEP1B	CSTL1
HPSE	MMP24
EGLN3	ADAMTS4
CSTB	FAM20C
ITIH5	MMP20
SERPINB7	TGM4
ADAMTSL3	SERPINB11
A2ML1	CST8
PRSS1	ADAMTS8
KNG1	MMP27
ADAM18	F7
AMBP	HTRA1
MMP12	CTSL
ADAMTSL2	SERPINA10
PAPPA2	CD109
TLL2	SERPINB6
SERPINA9	MMP9
ADAM33	SERPINA6
F13B	CTSH
ITIH3	SULF1
CST7	TGM7
SERPINB13	CST3
HPSE2	HABP2
ADAMTS14	TIMP3
CELA3A	PLAU
ST14	LOXL1
ADAMTS15	SERPING1
LOXL3	SERPINA11
TGM5	HTRA3
F10	MMP2
CELA2B	PLAT
SERPINB5	TIMP4
MMP11	SULF2
CST11	

EMS404

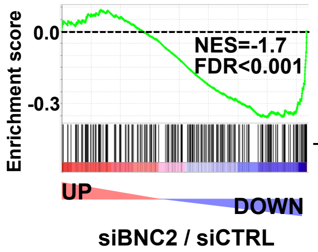

ADAMTS12
SERPINI1
ADAM23
CTSB
MMP16
P4HA1
CTSZ
PRSS12
P4HA2
ADAMTSL4
CELA3B
ADAM29
TIMP4
KAZALD1
SERPINH1
CST7
ADAM10
SERPINB3
MMP1
CTSA
PLOD1
LOX
NGLY1
PAPPA2
ADAM17
PLAT
SERPINF1
LOXL1
SERPINB5
HYAL3
CTSD
ADAMTS2
SERPINE1
MMP11
SERPING1
ADAM12
ADAMTS1
ADAM33
MASP1
HTRA3
PLOD2
TGM2
MMP14
LEPREL1
SERPINB1
LEPRE1
P4HA3
ADAMTS5
MMP13
ADAMTSL1
SERPIND1

Supplementary Fig.15. BNC2 controls expression of both ECM constituents and regulators.

A. Enrichment plots from GSEA characterizing transcriptomic changes induced by siBNC2 in LX2 cells (left panels) or EMS404 cells (right panels) compared to cells transfected with a siCTRL (control). A selective subset of the matrisome gene set used in Fig.7C-D was used corresponding to collagens (n=44 genes. **B.** Similar analyses using a selective subset of the matrisome gene set used in Fig.7C-D corresponding to ECM regulators (n=288 genes).

In all panels, NES and FDR are the normalized enrichment score and the false discovery rate provided by GSEA, respectively. The subset of genes which in each independent analysis accounts for the biased distribution of the gene set (GSEA core enrichment) is provided with similar genes between LX2 and EMS404 cells highlighted in blue.

Fig.S16

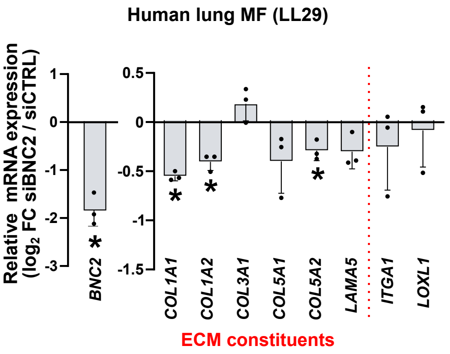

Human lung MF (LL29)
Relative mRNA expression
(log2 FC siBNC2 / siCTRL)
1
0
-1
-2
-3
0.5
0
-0.5
-1
-1.5
*
*
*
*
BNC2
COL1A1
COL1A2
COL3A1
COL5A1
COL5A2
LAMA5
ITGA1
LOXL1
ECM constituents

Supplementary Fig.16. BNC2 is expressed and functional in lung MFs.

RT-qPCR data showing the changes in expression of *BNC2* and indicated matrisome genes upon BNC2 silencing in human LL29 lung MFs. Cells were transfected with *BNC2*-targeting siRNA (siBNC2) or a non-targeting control siRNA (siCTRL) for 48h (n=3 biologically independent experiments). Log_2 FC between siBNC2 and siCTRL transfected cells are shown. The bar graph shows means ±SD. Two-sided one-sample t-test with Benjamini-Hochsberg correction for multiple testing was used to determine if the mean log_2 FC was statistically different from 0. * $p<0.05$, ** $p<0.01$, *** $p<0.001$.

Fig.S17

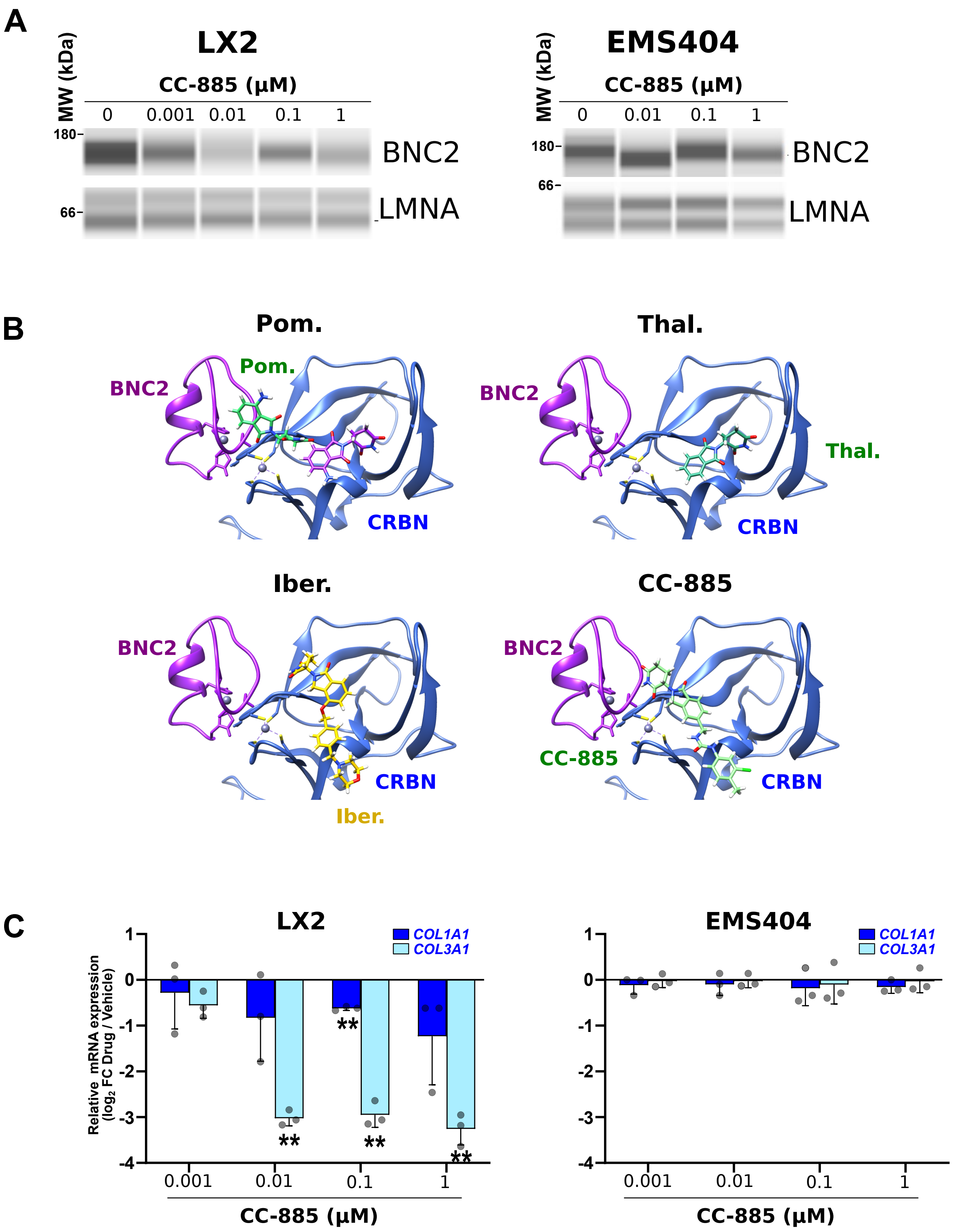

A
LX2
EMS404
MW (kDa)
CC-885 (µM)
0
0.001
0.01
0.1
1
180
66
BNC2
LMNA
B
Pom.
Thal.
Iber.
CC-885
BNC2
CRBN
C
LX2
EMS404
COL1A1
COL3A1
Relative mRNA expression (log2 FC Drug / Vehicle)
**
CC-885 (µM)

Supplementary Fig.17. CC-885 decreases BNC2 protein levels in human MF-HSCs but not murine MF-HSCs.

A. BNC2 protein expression levels in LX2 cells (left panel) or EMS404 cells (right panel) treated with increasing concentrations of CC-885 or vehicle for 24h were analyzed using Simple Western immunoassays. Antibodies directed against BNC2 or LMNA (control loading protein) were used. Shown images are representative of those obtained for 3 biologically independent experiments. MW, molecular weight markers. **B.** Docking of thalidomide and its derivatives on the BNC2 zinc finger 1 (violet)-CRBN (blue) structural model. Docking is shown for pomalidomide, thalidomide, iberdomide which was similar in size to CC-885, and finally CC-885. Pomalidomide (green) is shifted away from the zinc finger and not forming any interactions with it, suggesting it may not help the binding of BNCZF1 to CRBN. To assess the validity of this result, we docked in the same conditions pomalidomide into the original IKZF1-CRBN complex (purple). While there was not a perfect superimposition of the 30 solutions, all the poses were forming a single cluster in the original position, with no shift away from the zinc finger, therefore validating the docking protocol and pointing to a clear difference of effect of pomalidomide as a function of the zinc finger in the complex. Thalidomide (green) formed a closely superimposed cluster exactly comparable to pomalidomide. Iberdomide (yellow) was not correctly superimposed, with only 6 poses forming a close cluster once again far from zinc finger and rather well superimposed with pomalidomide. CC-885 (green), in its S configuration, was superimposed in a conformation where the piperidinedione spread toward the zinc finger and made contacts with both BNC2ZF1 and CRBN. Additional hydrogen bonds between CC-885 and CRBN were also predicated by the docking further suggesting that CC-885 may form a bridge between BNCZF1 and CRBN. **C.** RT-qPCR data showing expression of *COL1A1* and *COL3A1* upon treatment of LX2 cells (left panel) or EMS404 cells (right panel) with increasing concentrations of CC-885 or vehicle for 24h (n=3 biologically independent experiments). Log_2 FC between drug and vehicle-treated cells are shown. The bar graph shows means ±SD. Two-sided one-sample t-test with Benjamini-Hochsberg correction for multiple testing was used to determine if the mean log_2 FC was statistically different from 0. ** $p<0.01$.

Fig.S18

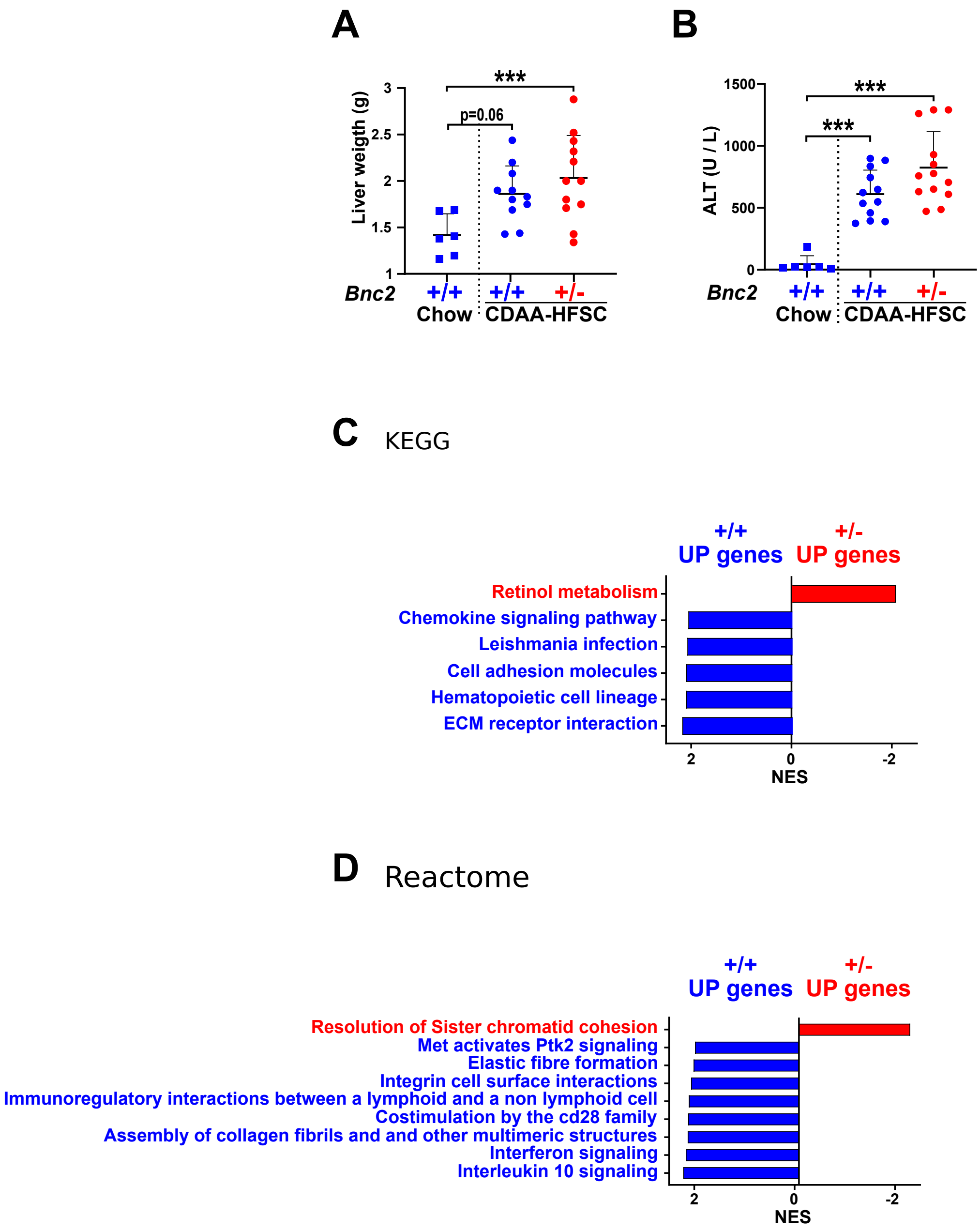
A
Liver weigth (g)
3
2.5
2
1.5
1
***
p=0.06
Bnc2
+/+
+/+
+/-
Chow
CDAA-HFSC
B
ALT (U / L)
1500
1000
500
0
***
***
Bnc2
+/+
+/+
+/-
Chow
CDAA-HFSC
C
KEGG
+/+
UP genes
+/-
UP genes
Retinol metabolism
Chemokine signaling pathway
Leishmania infection
Cell adhesion molecules
Hematopoietic cell lineage
ECM receptor interaction
2
0
-2
NES
D
Reactome
+/+
UP genes
+/-
UP genes
Resolution of Sister chromatid cohesion
Met activates Ptk2 signaling
Elastic fibre formation
Integrin cell surface interactions
Immunoregulatory interactions between a lymphoid and a non lymphoid cell
Costimulation by the cd28 family
Assembly of collagen fibrils and and other multimeric structures
Interferon signaling
Interleukin 10 signaling
2
0
-2
NES

Supplementary Fig.18. Additional characterization of *Bnc2* heterozygous mice fed the CDAA-HFSC diet.

A. Livers from heterozygous mice (+/-, n=12 mice) and WT mice (+/+, n=11 mice) fed for 7.5 weeks with the CDAA-HFSC diet together with livers from control WT mice fed a chow diet (n=6 mice) were collected the day of sacrifice and weighted. **B.** Blood from heterozygous mice (+/-, n=13 mice) and WT mice (+/+, n=12 mice) fed for 7.5 weeks with the CDAA-HFSC diet together with livers from control WT mice fed a chow diet (n=6 mice) were collected the day of sacrifice and used to measure alanine aminotransferase (ALT) activity. In A and B, the graphs show means ±SD. Statistical significance was assessed using one-way Anova with Tukey multiple comparison post-hoc test. *** p<0.001. **C-D.** Enrichment analysis of differentially expressed genes between livers of *Bnc2*$^{+/-}$ (n=10 mice) and Bnc2$^{+/+}$ (n=11 mice). GSEA was performed using transcriptomic changes induced by *Bnc2* deficiency as the ranked gene list and KEGG (C) or Reactome (D) pathways (MiSigDB, v7.2) as gene sets. Normalized Enrichment Scores (NES) for terms with FDR<10^{-4} are shown.

Fig.S19

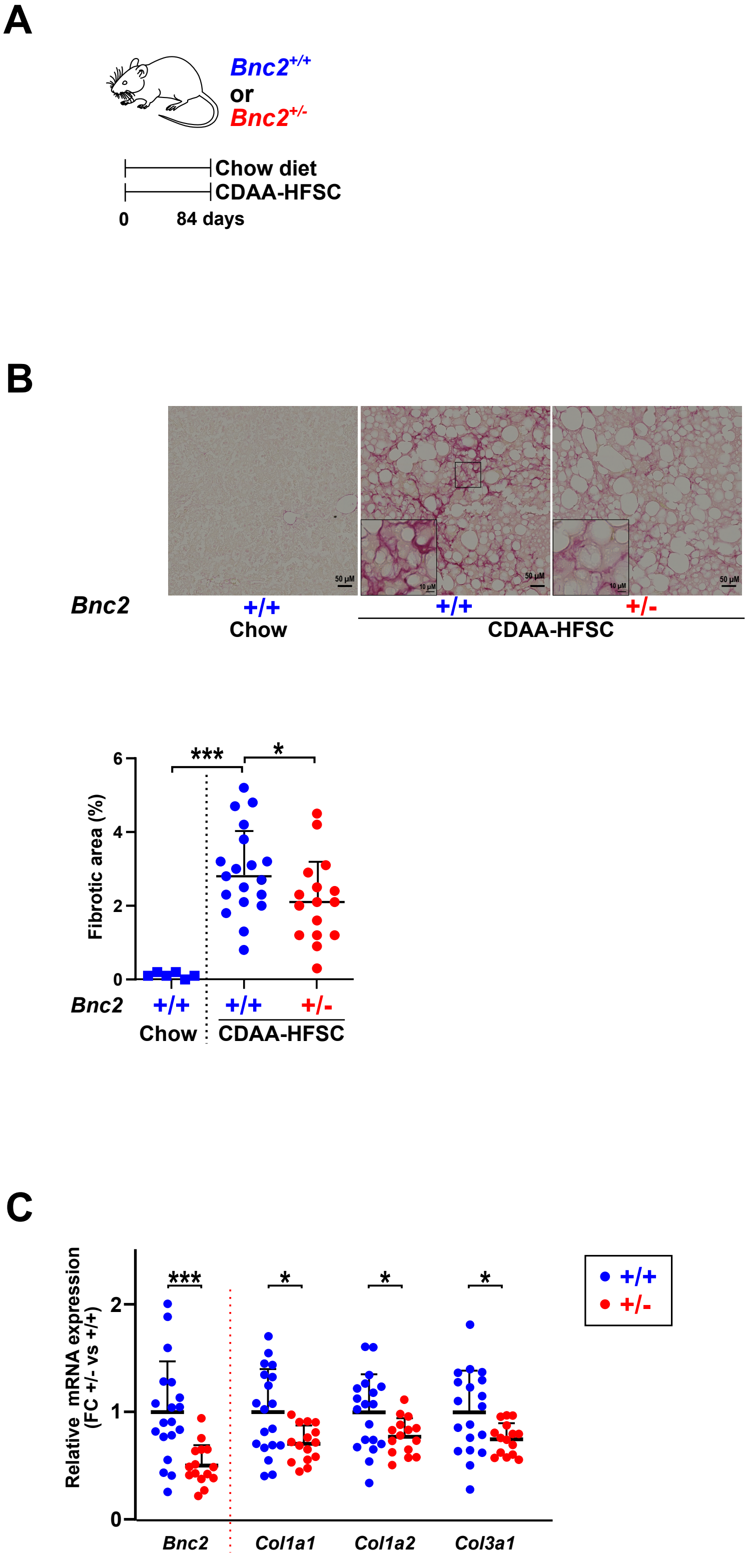
A
Bnc2+/+
or
Bnc2+/-
Chow diet
CDAA-HFSC
0
84 days
B
50 μM
10 μM
Bnc2
+/+
Chow
+/+
+/-
CDAA-HFSC
***
*
Fibrotic area (%)
6
4
2
0
Bnc2
+/+
+/+
+/-
Chow
CDAA-HFSC
C
Relative mRNA expression
(FC +/- vs +/+)
2
1
0
***
*
*
*
Bnc2
Col1a1
Col1a2
Col3a1
+/+
+/-

Supplementary Fig.19. *Bnc2* deficiency dampens collagen deposition in the livers of mice fed the pro-fibrotic CDAA-HFSC diet for an extended period of 12 weeks.

A. Schematic summary of the experimental protocol. Heterozygous mice (+/-, n=16 mice) and WT mice (+/+, n=19 mice) were fed the CDAA-HFSC diet for 12 weeks. Control WT mice were fed a chow diet (n=6 mice). Blood and livers were collected the day of sacrifice for biochemistry and histological analyses. A mouse was depicted using "Vector diagram of laboratory mouse (black and white)", https://commons.wikimedia.org/wiki/File:Vector_diagram_of_laboratory_mouse_(black_and_white).svg, available under the Creative Commons Attribution-Share Alike 4.0 International license (https://creativecommons.org/licenses/by-sa/4.0/deed.en). **B.** Quantification of collagen deposition was performed using Sirius red staining of two sections per liver from mice described in A. Entire liver sections were used for quantification. The top right panel shows representative images obtained for WT (+/+) or heterozygous (+/-) mice. One-way Anova with Tukey multiple comparison post-hoc test was used to assess statistical significance of observed differences. **C.** RT-qPCR data showing the expression of *Bnc2* and collagen encoding genes in WT (+/+; n=19 mice) and heterozygous (+/-; n=15 mice) mice. One-tailed Mann-Whitney U-test with Benjamini-Hochsberg correction for multiple testing was used to assess statistical significance of observed differences. In panels B-C, bars depict mean ±SD. Statistical significance is shown as follow * $p<0.05$, ** $p<0.01$, *** $p<0.001$.

Fig.S20

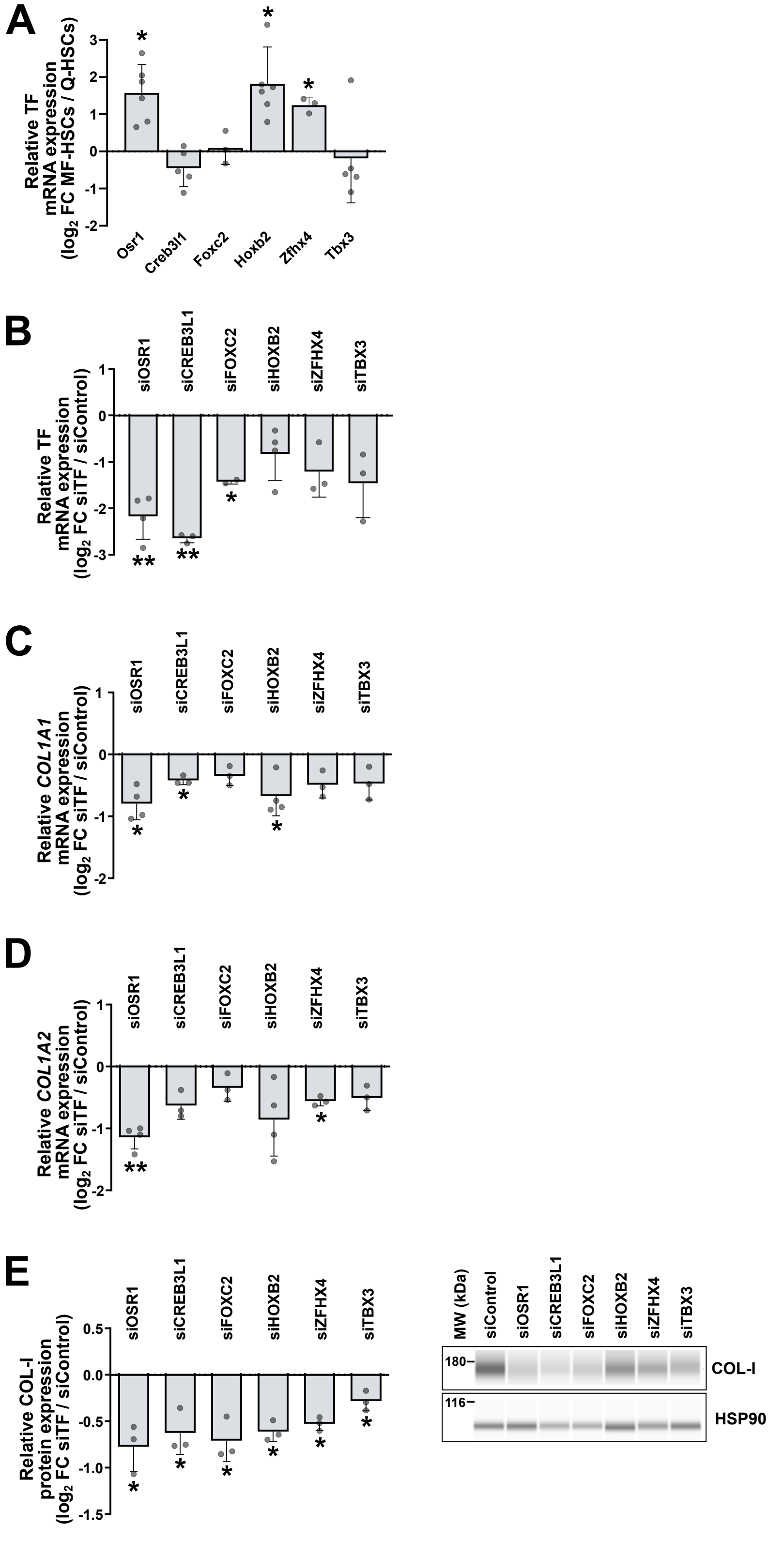
A
Relative TF mRNA expression (log2 FC MF-HSCs / Q-HSCs)
Osr1
Creb3l1
Foxc2
Hoxb2
Zfhx4
Tbx3
B
Relative TF mRNA expression (log2 FC siTF / siControl)
siOSR1
siCREB3L1
siFOXC2
siHOXB2
siZFHX4
siTBX3
C
Relative COL1A1 mRNA expression (log2 FC siTF / siControl)
D
Relative COL1A2 mRNA expression (log2 FC siTF / siControl)
E
Relative COL-I protein expression (log2 FC siTF / siControl)
MW (kDa)
siControl
180
116
COL-I
HSP90

Supplementary Fig.20. Silencing of the predicted MF identity TF OSR1, CREB3L1, FOXC2, HOXB2, ZFHX4 and TBX3 triggers reduced MF expression of type I collagen.

A. RT-qPCR data showing the changes in expression of indicated TFs upon murine primary quiescent HSCs (Q-HSCs) spontaneous *in vitro* activation into MF-HSCs [n=3 (*Foxc2*, *Zfhx4*), 5 (*Creb3l1*, *Tbx3*) or 6 (*Osr1*, *Hoxb2*)] biologically independent replicates]. Log_2 fold changes (FC) between MF-HSCs (6 days of culture) and Q-HSCs (1 day of culture) are shown. **B-D.** EMS404 cells were transfected with a siRNA targeting *Osr1* (siOSR1), *Creb3l1* (siCREB3L1), *Foxc2* (siFOXC2), *Hoxb2* (siHOXB2), *Zfhx4* (siZFHX4), *Tbx3* (siTBX3) or a non-targeting control siRNA (siCTRL) for 48h. Data are from biologically independent replicates: panel B [n=2 (siFOXC2), 3 (siCREB3L1, siZFHX4, siTBX3) or 4 (siOSR1, siHOXB2)]; panel C [n=3 (siCREB3L1, siFOXC2, siZFHX4, siTBX3) or 4 (siOSR1, siHOXB2)]; panel D [n=3 (siCREB3L1, siFOXC2, siZFHX4, siTBX3) or 4 (siOSR1, siHOXB2)]. Log_2 fold changes (FC) between MF-HSCs transfected with the indicated siTF and MF-HSCs transfected with siCTRL are shown. Bar graphs show expression data for the different TFs (i.e. validation of silencing efficacy; panel B), for *Col1a1* (panel C) or for *Col1a2* (panel D). **E.** Type I Collagen (COL-I) protein expression in EMS404 cells transfected with the indicated siTF. Total protein extracts were subjected to Simple Western immunoassay using antibody against type I collagen (COL-I). HSP90 are used as protein loading control. The left panel show the results of protein quantification obtained from 3 independent experiments. MW, molecular weight markers.

For all panels, the bar graphs show means ±SD. Two-sided one-sample t-test with Benjamini-Hochsberg correction for multiple testing was used to determine if the mean log_2 FC was statistically different from 0. * $p<0.05$, ** $p<0.01$.

Fig.S21

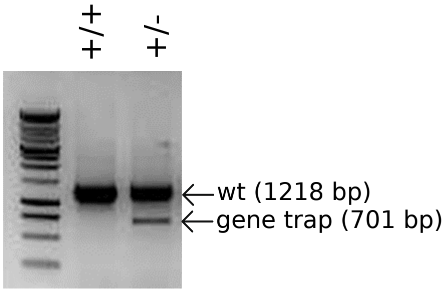

Supplementary Fig.21. Validation of the *Bnc2* heterozygous genotype.

Genomic DNA were extracted from mouse tails and multiplex PCR was performed as described in [11]. The presence of an amplicon at 701 bp indicates the presence of the transgene in +/- mice. The presented image is representative of those obtained throughout several years of Ayu21-18 mice genotyping.

SUPPLEMENTARY REFERENCES

1. Higuchi, Y. *et al.* Gastrointestinal Fibroblasts Have Specialized, Diverse Transcriptional Phenotypes: A Comprehensive Gene Expression Analysis of Human Fibroblasts. *PLoS One* **10**, e0129241 (2015).
2. Terkelsen, M. K. *et al.* Transcriptional dynamics of hepatic sinusoid-associated cells after liver injury. *Hepatology* (2020) doi:10.1002/hep.31215.
3. Reyfman, P. A. *et al.* Single-Cell Transcriptomic Analysis of Human Lung Provides Insights into the Pathobiology of Pulmonary Fibrosis. *Am J Respir Crit Care Med* **199**, 1517–1536 (2019).
4. Fu, X. *et al.* Specialized fibroblast differentiated states underlie scar formation in the infarcted mouse heart. *J Clin Invest* **128**, 2127–2143 (2018).
5. Guilliams, M. *et al.* Spatial proteogenomics reveals distinct and evolutionarily conserved hepatic macrophage niches. *Cell* **185**, 379-396.e38 (2022).
6. Vandel, J. *et al.* Hepatic Molecular Signatures Highlight the Sexual Dimorphism of Nonalcoholic Steatohepatitis (NASH). *Hepatology* **73**, 920–936 (2021).
7. Buckley, M. A. *et al.* Functional Analysis and Fine Mapping of the 9p22.2 Ovarian Cancer Susceptibility Locus. *Cancer Res.* **79**, 467–481 (2019).
8. Martin-Malpartida, P. *et al.* Structural basis for genome wide recognition of 5-bp GC motifs by SMAD transcription factors. *Nat Commun* **8**, 2070 (2017).
9. McLean, C. Y. *et al.* GREAT improves functional interpretation of cis-regulatory regions. *Nat. Biotechnol.* **28**, 495–501 (2010).
10. Hait, T. A., Amar, D., Shamir, R. & Elkon, R. FOCS: a novel method for analyzing enhancer and gene activity patterns infers an extensive enhancer–promoter map. *Genome Biol* **19**, 56 (2018).
11. Vanhoutteghem, A. *et al.* Basonuclin 2 has a function in the multiplication of embryonic craniofacial mesenchymal cells and is orthologous to disco proteins. *Proc. Natl. Acad. Sci. U.S.A.* **106**, 14432–14437 (2009).
