# Supplementary material for: Functional genomics uncovers the transcription factor BNC2 as required for myofibroblastic activation in fibrosis
Source: Nat Commun. 2022 Sep 10;13:5324. doi: 10.1038/s41467-022-33063-9 (PMC9464213; doi:10.1038/s41467-022-33063-9)
Supplement: Supplementary file 3 — Description of Additional Supplementary Files [file 41467_2022_33063_MOESM3_ESM.pdf]

Description of Additional Supplementary Files

File Name: Supplementary Data 1
Description: Genes and TF encoding genes associated with broad H3K4me3 in MFs

File Name: Supplementary Data 2
Description: Cells/tissues used to obtain the H3K4me3 ChIP-seq data used in Fig.1B

File Name: Supplementary Data 3
Description: Primary cells used to obtain the transcriptomics data used in Fig.1C

File Name: Supplementary Data 4
Description: Clinical characteristics of donors from the TargetOH cohort

File Name: Supplementary Data 5
Description: Clinical characteristics of donors from the ABOS cohort

File Name: Supplementary Data 6
Description: All proteins and peptides detected in BNC2 RIME data using antibody HPA018525 (Sigma-Aldrich) and non-immune IgG control

File Name: Supplementary Data 7
Description: Coordinates of BNC2 ChIP-seq peaks from LX2 cells (hg38)

File Name: Supplementary Data 8
Description: All proteins and peptides detected in BNC2 RIME data using antibody 55220-1-AP (Proteintech) and non-immune IgG controls

File Name: Supplementary Data 9
Description: Clustering of Molecular Function terms enriched for predicted BNC2 target genes

File Name: Supplementary Data 10
Description: Table of reagents

File Name: Supplementary Data 11
Description: Publicly available datasets used in this study
