# Supplementary material for: Functional genomics uncovers the transcription factor BNC2 as required for myofibroblastic activation in fibrosis
Source: Nat Commun. 2022 Sep 10;13:5324. doi: 10.1038/s41467-022-33063-9 (PMC9464213; doi:10.1038/s41467-022-33063-9)
Supplement: Supplementary file 15 — Reporting Summary [file 41467_2022_33063_MOESM15_ESM.pdf]

nature portfolio

Corresponding author(s): Jerome Eeckhoute

Last updated by author(s): 2022/08/16

Reporting Summary

Nature Portfolio wishes to improve the reproducibility of the work that we publish. This form provides structure for consistency and transparency in reporting. For further information on Nature Portfolio policies, see our Editorial Policies and the Editorial Policy Checklist.

Statistics

For all statistical analyses, confirm that the following items are present in the figure legend, table legend, main text, or Methods section.

n/a	Confirmed	
☐	☒	The exact sample size (*n*) for each experimental group/condition, given as a discrete number and unit of measurement
☐	☒	A statement on whether measurements were taken from distinct samples or whether the same sample was measured repeatedly
☐	☒	The statistical test(s) used AND whether they are one- or two-sided<br>*Only common tests should be described solely by name; describe more complex techniques in the Methods section.*
☐	☒	A description of all covariates tested
☐	☒	A description of any assumptions or corrections, such as tests of normality and adjustment for multiple comparisons
☐	☒	A full description of the statistical parameters including central tendency (e.g. means) or other basic estimates (e.g. regression coefficient) AND variation (e.g. standard deviation) or associated estimates of uncertainty (e.g. confidence intervals)
☐	☒	For null hypothesis testing, the test statistic (e.g. *F*, *t*, *r*) with confidence intervals, effect sizes, degrees of freedom and *P* value noted<br>*Give P values as exact values whenever suitable.*
☒	☐	For Bayesian analysis, information on the choice of priors and Markov chain Monte Carlo settings
☐	☒	For hierarchical and complex designs, identification of the appropriate level for tests and full reporting of outcomes
☒	☐	Estimates of effect sizes (e.g. Cohen's *d*, Pearson's *r*), indicating how they were calculated

Our web collection on statistics for biologists contains articles on many of the points above.

Software and code

Policy information about availability of computer code

Data collection

Provide a description of all commercial, open source and custom code used to collect the data in this study, specifying the version used OR state that no software was used.

Data analysis

All softwares and codes used for data analysis are open source and publicly available.
Data analyses were performed using:
aplpack v1.3.2 R package
bioDBnet
Bowtie 2 v1.0.0
Cistrome DB toolkit
Command Console v4.1.2 (Affymetrix)
Compass (ProteinSimple)
DeepTools v3.3.2
easyPubMed
EdgeR v0.0.3
FlowJo v10.5.3
FOCS
Galaxy
GeneSnap v7.12.06
GIANT v0.0.2
G-MAD
gplots v3.0.1
graphics R package
GREAT v2.0 (H3K4me3 ChIP-seq) or 4.0.4 (BNC2 ChIP-seq)
GSEA v3.0

Htseq-count v1.0.0
Image J version 1.53c
Image Studio Lite v5.2
Integrated Genome Browser (IGB 9.0.1)
MACS2 v2.1.1.20160309
Metascape
Prism v5 and 8
RSAT
String 11.0
Tophap v2.0.9
Seurat v4.0.3
ZEN v2.0.0.0
Additional details are provided in Supplementary Data file 10.

For manuscripts utilizing custom algorithms or software that are central to the research but not yet described in published literature, software must be made available to editors and reviewers. We strongly encourage code deposition in a community repository (e.g. GitHub). See the Nature Portfolio guidelines for submitting code & software for further information.

Data

Policy information about availability of data

All manuscripts must include a data availability statement. This statement should provide the following information, where applicable:

- Accession codes, unique identifiers, or web links for publicly available datasets
- A description of any restrictions on data availability
- For clinical datasets or third party data, please ensure that the statement adheres to our policy

Transcriptomic and cistromic data generated in this study have been deposited into Gene Expression Omnibus under SuperSeries accession number GSE185529 [https://www.ncbi.nlm.nih.gov/geo/query/acc.cgi?acc=GSE185529]. The mass spectrometry proteomics data generated in this study have been deposited to the ProteomeXchange Consortium via the PRIDE [1] partner repository with the dataset identifier PXD003624 [https://www.ebi.ac.uk/pride/archive/projects/PXD003624]. Datasets analyzed in this study (Supplementary Data file 11) are available through the Gene Expression Omnibus (GSE68108 [https://www.ncbi.nlm.nih.gov/geo/query/acc.cgi?acc=GSE68108], GSE58680 [https://www.ncbi.nlm.nih.gov/geo/query/acc.cgi?acc=GSE58680], GSE38103 [https://www.ncbi.nlm.nih.gov/geo/query/acc.cgi?acc=GSE38103], GSE61852 [https://www.ncbi.nlm.nih.gov/geo/query/acc.cgi?acc=GSE61852], GSE68108 [https://www.ncbi.nlm.nih.gov/geo/query/acc.cgi?acc=GSE68108], GSE111059 [https://www.ncbi.nlm.nih.gov/geo/query/acc.cgi?acc=GSE111059], GSE63626 [https://www.ncbi.nlm.nih.gov/geo/query/acc.cgi?acc=GSE63626], GSE145086 [https://www.ncbi.nlm.nih.gov/geo/query/acc.cgi?acc=GSE145086], GSE192742 [https://www.ncbi.nlm.nih.gov/geo/query/acc.cgi?acc=GSE192742]), ENCODE (https://www.encodeproject.org/experiments/ENCSR507UDH/), FANTOM5 (https://fantom.gsc.riken.jp/5/sstar), the matrisome database (http://matrisomeproject.mit.edu/ and http://matrisomedb.pepchem.org/), and manuscript supplementary data (https://www.nature.com/articles/ng.3385 and https://www.atsjournals.org/doi/10.1164/rccm.201712-2410OC). Source data are provided with this paper.

Field-specific reporting

Please select the one below that is the best fit for your research. If you are not sure, read the appropriate sections before making your selection.

☒ Life sciences ☐ Behavioural & social sciences ☐ Ecological, evolutionary & environmental sciences

For a reference copy of the document with all sections, see nature.com/documents/nr-reporting-summary-flat.pdf

Life sciences study design

All studies must disclose on these points even when the disclosure is negative.

Sample size	The sample size was chosen as a function of the inherent variability of the different types of experiments/models. In vitro experiments (cell-lines or mouse primary HSCs) were repeated at least three times (independent biological experiments), with each experiment being performed in technical triplicates, based on experience and previous work (e.g. Lefebvre et al, JCI insight 2017, Dubois et al MolSystBio 2020). For in vivo experiments, the number of mice used in this study is based on the litterature. It has been shown that 10 to 15 mice are sufficient to show significant variation of the fibrosis level based on histological data (Pawlak et al., Hepatology, 2014; Lefevbre et al., JCI insight, 2017)
Data exclusions	No data were excluded from the analyses
Replication	The number of independent experiments are indicated in figure legends. Human primary hepatic stellate cells were from 3 different donors. Liver fibrosis development in Bnc2 heterozygous mice was assessed in two entirely independent experiments each including at least 12 mice. Differences in biological replicates between genes or panels stem from technical issues or shortage of biological material.
Randomization	The effects of subjective bias have been minimized. For in vitro experiments, all compared groups were treated and processed simulaneously within each round of replicates. For in vivo experiments, mice from different genotypes were mixed within the cages to insure randomization of the study.
Blinding	For fibrosis area measurement using Sirius red staining, scientists were blinded both with regards to sample preparation and analysis since they were not aware of the genotype of the mice. For other types of experiments, blinding was not relevant since absolute quantitative methods without human subjectivity were used.

Reporting for specific materials, systems and methods

We require information from authors about some types of materials, experimental systems and methods used in many studies. Here, indicate whether each material, system or method listed is relevant to your study. If you are not sure if a list item applies to your research, read the appropriate section before selecting a response.

Materials & experimental systems

n/a	Involved in the study
☐	☒ Antibodies
☐	☒ Eukaryotic cell lines
☒	☐ Palaeontology and archaeology
☐	☒ Animals and other organisms
☐	☒ Human research participants
☒	☐ Clinical data
☒	☐ Dual use research of concern

Methods

n/a	Involved in the study
☐	☒ ChIP-seq
☐	☒ Flow cytometry
☒	☐ MRI-based neuroimaging

Antibodies

Antibodies used

Primary antibodies :
ACTA2, Abcam, Cat# ab124964 (WB 1/1000, Flow cytometry dilution 1:50)
ACTB, Sigma-Aldrich, Cat # A5441 (Wes dilution 1:50)
BNC2, Sigma-Aldrich, Cat# HPA018525 (IP 2 µg, ChIP 3 µg, RIME 10 µg)
BNC2, Sigma-Aldrich, Cat# HPA059419 (Wes 1/50)
BNC2, Protein Tech, Cat# 55220-1-AP (IP 2 µg, RIME 10 µg)
BRD4, Wu S et al., Genes Dev 2006 N/A (Wes 1/50)
COL1A1 (Collagen I), Abcam, Cat# ab34710 (WB 1/1000, Wes 1/50)
DES, Abcam, Cat# ab32362 (Flow cytometry dilution 1:70)
GAPDH, Santa Cruz, Cat# sc-32233 (WB 1/4000)
H3, Cell Signaling, Cat# 4499 (WB 1/1000)
H3K27ac, Active motif, Cat# 39685 (ChIP 2 µg)
HSP90, Biolegend, Cat# 661802 (Wes 1/50, WB 1/5000)
LMNA (Lamin A/C), Santacruz Cat# sc-20681 (Wes 1/250, WB 1/2000)
YAP1, Cell signaling, Cat#14074T (Wes 1/50, WB 1/1000)

Secondary antibodies or control antibodies :
FITC-conjugated anti-rabbit Invitrogen, Cat# A11008 (Flow cytometry dilution 1:500)
HRP-conjugated anti-mouse, Sigma-Aldrich, Cat# A4416 (WB 1/5000)
HRP-conjugated anti-rabbit Sigma-Aldrich, Cat# A0545 (WB 1/5000)
Rabbit IgG (isotype control), Cell Signaling Cat# 2729 (IP 2 µg, RIME 10 µg, Flow cytometry dilution 1:50)

Validation

The primary antibodies presented above have been used and validated in previous studies (PMID30397120, PMID32407006, PMID34129887). Additional controls of specificity have been performed in our laboratory regarding myofibroblast markers (COL, DES, ACTA2; western blots, immunohistochemistry). Regarding anti-BNC2 antibodies, specific validations which have been performed for each application are presented in the manuscript and include:
For Western blotting experiments, cells were transfected with a siRNA targeting BNC2 or a BNC2 expression vector to validate antibody specificity (Fig. S6)
Initial RIME experiments were conducted and showed that BNC2 protein was specifically detected in the BNC2 RIME but not when using a non-immune IgG control antibody. This observation therefore validated the use of antibody HPA-018525 for immunoprecipitation-based experiments such as RIME and ChIP-seq

Eukaryotic cell lines

Policy information about cell lines

Cell line source(s)

Cell line sources are described in Table S8
EMS404 (M. musculus) Kerafast, Guo et al. Hepatology 2009, EMS404
LX2 (H. sapiens), Merck, scc064
LL29 (H. sapiens), Sigma-Aldrich, 87112508
AML-12 (M. musculus), ATCC CRL:2254
IHH (H. sapiens), Schippers et al. Cell Biol Toxicol 1997, N/A
Human primary MF-HSC (Donor 2, female), Samsara Sciences, HL180075 Don 2
Human primary MF-HSC (Donor 3, male), Innoprot, P10653 Don 3
Human primary MF-HSC (Donor 4, male), Cirrhotic liver obtained from Huriez Hospital's Liver Unit (Lille, France), N/A

Authentication

As the EMS404 cell line had not been thoroughly used in the literature, we verified proper expression of MF markers and their ability to respond to TGFβ (Fig. S11).

For human MF-HSC, the expression of MF specific marker such as Acta2 or Col1a1 was verified by immunofluorescence.
No specific procedure was used for the other cell lines

Mycoplasma contamination

Cells line were tested negative for mycoplasma using a PCR method performed every month

Commonly misidentified lines
(See ICLAC register)

No misidentified lines were used in this study.

Animals and other organisms

Policy information about studies involving animals; ARRIVE guidelines recommended for reporting animal research

Laboratory animals

Mice were housed in standard cages in a temperature-controlled room (22-24°C) with a 12-h dark-light cycle
For primary mouse isolation, male mice (C57BL/6J, 15-18 weeks old) were used.
For CCl4 studies, experiments were performed using female mice (C57BL/6J, 10-14 weeks old)
For studies using the HFSC diet, experiments were performed using male mice (C57BL/6J, 8 weeks old)
For studies using the CDAA-HFSC diet, experiments were performed using Ayu21-18 (Bnc2+/- mice) and WT littermate male mice (C57Bl/6xCBA, 13-19 weeks old)

Wild animals

The study did not involve wild animals.

Field-collected samples

The study did not involve samples collected from the field.

Ethics oversight

All animal studies were performed in compliance with EU specifications regarding the use of laboratory animals and have been approved by the Nord-Pas de Calais Ethical Committee (APAFIS#15539-2018053011323354).

Note that full information on the approval of the study protocol must also be provided in the manuscript.

Human research participants

Policy information about studies involving human research participants

Population characteristics

Clinical parameters of the patients are described in Table S4 (TargetOH cohort) and Table S5 (ABOS cohort)

Recruitment

Donors from the ABOS cohort were recruited among obese patients visiting the Obesity Surgery Department
at the Centre Hospitalier Universitaire de Lille (Lille, France). All patients fulfilled criteria for, and were willing to undergo, weight-loss surgery. Greater details were provided previously (PMID: 32394476).
Regarding the TargetOH cohort, patients with clinical and biological characteristics of alcoholic steatohepatitis or cirrhosis not responding to medical therapy have been selected in the Lille University Hospital (Lille, France) according to previously published selection criteria (PMID25731872; PMID34129887).

Ethics oversight

Study is authorized by the Lille ethical committee and informed consent was obtained from all subjects.

Note that full information on the approval of the study protocol must also be provided in the manuscript.

ChIP-seq

Data deposition

☒ Confirm that both raw and final processed data have been deposited in a public database such as GEO.

☒ Confirm that you have deposited or provided access to graph files (e.g. BED files) for the called peaks.

Data access links
May remain private before publication.

https://www.ncbi.nlm.nih.gov/geo/query/acc.cgi?acc=GSE185529

Files in database submission

LX2_siCTRL_1.CEL
LX2_siCTRL_2.CEL
LX2_siCTRL_3.CEL
LX2_siCTRL_4.CEL
LX2_siBNC2_1.CEL
LX2_siBNC2_2.CEL
LX2_siBNC2_3.CEL
LX2_siBNC2_4.CEL
LX2 siBNC2 normalized expression tables
EMS404_siCTRL_1.CEL
EMS404_siCTRL_2.CEL
EMS404_siCTRL_3.CEL
EMS404_siCTRL_4.CEL
EMS404_siBNC2_1.CEL
EMS404_siBNC2_2.CEL
EMS404_siBNC2_3.CEL

EMS404_siBNC2_4.CEL
EMS404 siBNC2 normalized expression tables
LX2_BNC2_ChIPseq.fastq
LX2_BNC2_ChIPseq_INPUT.fastq
LX2_H3K27ac_ChIPseq.fastq
LX2_H3K27ac_ChIPseq_INPUT.fastq
LX2_CoP-seq.fastq
LX2_CoP-seq_INPUT.fastq
LX2_BNC2_ChIPseq.bigwig
LX2_H3K27ac_ChIPseq.bigwig
LX2_CoP-seq.bigwig
LX2_BNC2_peaks.bed
WT_1.CEL
WT_2.CEL
WT_3.CEL
WT_4.CEL
WT_5.CEL
WT_6.CEL
WT_7.CEL
WT_8.CEL
WT_9.CEL
WT_10.CEL
Bnc2Het_1.CEL
Bnc2Het_2.CEL
Bnc2Het_3.CEL
Bnc2Het_4.CEL
Bnc2Het_5.CEL
Bnc2Het_6.CEL
Bnc2Het_7.CEL
Bnc2Het_8.CEL
Bnc2Het_9.CEL
Bnc2Het_10.CEL
Bnc2 heterozygous and WT mice under CDAA-HFSC normalized expression tables

Genome browser session (e.g. UCSC)

BigWig files provided in GEO can be used to visualize data in any genome browser

Methodology

Replicates

Transcriptomics analyses involved 4 independent replicates for cell-line experiments and 10 mice per group for in vivo experiments. We sequenced one replicate of ChIP or CoP together with the corresponding inputs. Additional ChIP-qPCR were run on independent samples.

Sequencing depth

For each experiment, Illumina Next-seq 500 were used with 60 million reads (Single-end, 75 bp)

Antibodies

BNC2 antibody (Sigma, HPA018525, lot A45570)
H3K27ac (#39685, Active Motif, lot 31416013)

Peak calling parameters

ChIP-seq data were processed using a local instance of Galaxy (Afgan et al., 2018). Genome-wide signal tracks and enriched regions (peak calling) were obtained using model-based analysis of ChIP-seq version 2 (MACS2 v2.1.1.20160309). Input DNA was used as control, duplicate tags and those mapping to ENCODE blacklisted regions v2 15 were removed. A length of 300 bp was used as set extension size and the cutoff for peak detection was set to p<0.001. Integrated Genome Browser (IGB v9.0.1) was used to select the most relevant cut-off.
For H3K4me3 ChIP-seq processing, peak calling was performed using the broad option for histone marks (q<0.001)

Data quality

Data quality was assessed by FastQC analysis (http://www.bioinformatics.babraham.ac.uk/projects/fastqc). For BNC2 ChIP-seq, 1479 binding sites were retrieved with p-val<0.0001. Motif search and overlap between BNC2 peaks and H3K27ac ChIP-seq as well as CoP-seq was verified.

Software

ChIP-seq data were processed using a local instance of Galaxy (Afgan et al., 2018). Data processing involved FastQC analysis (http://www.bioinformatics.babraham.ac.uk/projects/fastqc) and read mapping to hg38 using Bowtie2 version 1.0.0. Genome-wide signal tracks and enriched regions (peak calling) were obtained using model-based analysis of ChIP-seq version 2 (MACS2 v2.1.1.20160309). ChIP-seq data have been deposited into Gene Expression Omnibus under SuperSeries accession number GSE185529

Flow Cytometry

Plots

Confirm that:

☒ The axis labels state the marker and fluorochrome used (e.g. CD4-FITC).

☒ The axis scales are clearly visible. Include numbers along axes only for bottom left plot of group (a 'group' is an analysis of identical markers).

☒ All plots are contour plots with outliers or pseudocolor plots.

☒ A numerical value for number of cells or percentage (with statistics) is provided.

Methodology

Sample preparation	Primary mouse HSCs were purified from C57BL/6J mice (male, 15-18 weeks old). Briefly, livers were digested in situ with 14 mg pronase (Sigma-Aldrich) and 3.7 U collagenase D (Roche) followed by in vitro digestion with 0.5 mg/mL pronase, 0.088 U/mL collagenase D and 0.02 mg/mL DNase I (Roche). HSCs were then separated by a Nycodenz gradient before sorting
Instrument	FACS Aria II SORP (BD Biosciences)
Software	FlowJo software was used for data analysis
Cell population abundance	The purity of HSCs was assessed by measuring the percentage of ultraviolet (UV; retinol autofluorescence) and Desmin-positive cells
Gating strategy	Cell debris were first removed based on FSC/SSC data. HSC were then defined as UV positive and Desmin positive cells with or without sorting

☒ Tick this box to confirm that a figure exemplifying the gating strategy is provided in the Supplementary Information.
